# Validating the Korean versions of the flourish index and the secure flourish index: a comprehensive psychometric approach

**DOI:** 10.3389/fpsyg.2026.1694272

**Published:** 2026-02-11

**Authors:** Jaeyoung Ha, Dorota Weziak-Bialowolska, Yoon Young Choi, Soong-nang Jang, Jieun Hwang, Sung-il Cho

**Affiliations:** 1 Graduate School of Public Health, Seoul National University, Seoul, Republic of Korea; 2 Department of Quantitative Methods and Information Technology, Kozminski University, Warsaw, Poland; 3 Human Flourishing Program, Institute for Quantitative Social Science, Harvard University, Cambridge, MA, United States; 4 Institute for Community Care and Health Equity, Chung-Ang University, Seoul, Republic of Korea; 5 Red Cross College of Nursing, Chung-Ang University, Seoul, Republic of Korea; 6 Department of Health Administration, College of Health Science, Dankook University, Cheonan, Republic of Korea; 7 Institute of Convergence Healthcare, Dankook University, Cheonan, Republic of Korea; 8 Institute of Health and Environment, Seoul National University, Seoul, Republic of Korea

**Keywords:** human flourishing, item response theory, multidimensional well-being, psychometrics, surveys and questionnaires

## Abstract

**Background:**

Human flourishing is conceptualized as a state of complete well-being encompassing core dimensions of life: (D1) happiness and life satisfaction, (D2) physical and mental health, (D3) meaning and purpose, (D4) character and virtue, and (D5) close social relationships, and one sustaining domain, (D6) financial and material stability. As an indicator that enables policy debates on redefining priorities around what constitutes a better life, developing and validating Korean translations of this measure is essential for meaningful international comparison. Therefore, this study aims to validate the Korean versions of the Flourish Index (FI) and the Secure Flourish Index (SFI), providing psychometric evidence and culturally grounded insights into the multidimensional construct of flourishing.

**Methods:**

A total of 1,217 adults participated in an online survey conducted between January 30 and February 8, 2024. To ensure comprehensive validation, reliability and structural validity were examined using classical test theory, while item-level functioning was evaluated using unidimensional and multidimensional graded response models within an item response theory framework.

**Results:**

The findings supported the conceptual and structural validity of both indices while indicating multidimensionality. Specifically, three domains (happiness and life satisfaction, mental health, and meaning and purpose) clustered closely and primarily contributed to the general flourishing factor. In contrast, character and virtue and close social relationships formed a distinct cluster, implying a potential personal–relational distinction. Items in the character and virtue domain showed signs of social desirability bias, while redundancy was noted between items in the close social relationships domain. Measurement invariance was supported at the configural and metric levels, and partially at the scalar level, and no significant differential item functioning was detected.

**Conclusion:**

These findings imply that culturally sensitive adaptations may enhance the interpretability and applicability of the instruments in cross-national research, thereby contributing to a more nuanced understanding of flourishing across diverse cultural contexts.

## 1 Introduction

Human flourishing refers to “a state in which all aspects of a person’s life are good” (VanderWeele, 2017). It encompasses not only happiness and health but also meaning and purpose, character and virtue, close social relationships, and security. Unlike conventional well-being measures, which often focus on a limited set of dimensions, such as subjective happiness, life satisfaction, or mental health, flourishing is inherently multidimensional. It integrates both hedonic and eudaimonic aspects of life with moral and relational components (VanderWeele and Johnson, 2025a). A key distinction between human flourishing and other subjective well-being measures lies in the explicit incorporation of both health and virtue into a comprehensive framework of well-being (VanderWeele, 2017). First, by including health as an essential component of a good life, flourishing extends the role of public health beyond disease prevention and life prolongation to the broader goal of promoting a better society and human flourishing in its fullest sense. Second, by incorporating virtue, such as civic character and prosociality, it moves beyond affective states to encompass moral values and contributions to the common good within the well-being framework. Furthermore, a substantial body of research indicates that individual components of flourishing (e.g., life satisfaction and meaning in life) are associated with a range of health outcomes, including healthier behaviors (Kim et al., 2021), reduced risk of cardiovascular disease (Kubzansky et al., 2018), and lower all-cause mortality (Cohen et al., 2016; Martín-María et al., 2017).

Another significant contribution of the human flourishing framework is that it provides a practical and psychometrically grounded tool for assessing an otherwise abstract and multidimensional construct. The Flourish Index (FI) and its extended form, the Secure Flourish Index (SFI), exemplify this approach by operationalizing these multidimensional concepts into concise, psychometrically robust domains that can be assessed across diverse cultural contexts and in longitudinal research (Wȩiak-Białowolska et al., 2019a; VanderWeele et al., 2025). The FI assesses five core domains, i.e., happiness and life satisfaction, mental and physical health, meaning and purpose, character and virtue, and close social relationships, while the SFI adds a sixth domain, financial and material stability. Both indices have been validated in multiple languages and are widely adopted in large-scale international studies, including the Global Flourishing Study (Lomas et al., 2025; VanderWeele et al., 2025).

Subjective well-being in South Korea presents distinctive sociocultural characteristics. Despite the country’s advanced economic development, levels of life satisfaction and subjective well-being remain comparatively low (OECD, 2024). At the same time, indicators of social vitality, such as satisfaction with social relationships, civic engagement, and interpersonal trust, have declined, while the proportion of single-person households and older adults living alone continues to rise (Statistics Korea, 2024). In many East Asian cultures, including Korea, happiness is often understood in interdependent terms, emphasizing relational harmony with family and close others (Hitokoto and Takahashi, 2021; Krys et al., 2023). These complex patterns imply that individual health and well-being are closely intertwined with broader sociocultural and structural factors. A comprehensive well-being framework can therefore offer an integrative lens through which to understand and address these multidimensional challenges. However, while health-related quality of life instruments are frequently used in Korean health research to assess well-being in relation to specific health conditions (Jo et al., 2017), tools that comprehensively capture physical, psychological, moral, and relational dimensions of well-being remain scarce. Consequently, validated Korean versions of the FI and SFI are limited. A previous study used a Korean translation of these instruments to examine university students’ flourishing during the COVID-19 pandemic (Jang et al., 2025); however, scale validation was not the primary objective of that study, and a comprehensive psychometric evaluation was not performed. This lack of rigorous validation limits the ability to monitor flourishing in the Korean context and constrains meaningful contributions to cross-cultural comparisons.

The present study translated the FI and SFI into Korean and evaluated their psychometric properties through a thorough examination. We used a comprehensive analytic strategy that integrated classical test theory (CTT) and item response theory (IRT). By combining these complementary frameworks, this study leverages the strengths of both: CTT provides robust evaluations of overall reliability, factor structure, and construct validity, while IRT offers fine-grained insights into item characteristics, including item discrimination, difficulty parameters, and measurement precision across the latent trait continuum. This integrated approach enables a more nuanced and rigorous evaluation of the scales, ensuring both psychometric soundness and practical applicability. This study provides the first validated Korean versions of the FI and SFI, delivering robust tools for monitoring and advancing research on human flourishing in Korea and facilitating their inclusion in cross-national investigations.

## 2 Materials and methods

### 2.1 Measures

#### 2.1.1 FI and SFI

The FI is a 10-item instrument designed to assess human flourishing. It comprises 10 items covering five domains, including happiness and life satisfaction, mental and physical health, meaning and purpose, character and virtue, and close social relationships, each represented by two items. The SFI extends the FI by including two additional items within the financial and material stability domain, resulting in a 12-item measure (Table 1). This added domain captures the stability of material and financial resources that sustain other aspects of flourishing (VanderWeele, 2017). All items are rated on an 11-point Likert-type scale ranging from 0 to 10, with higher scores indicating greater flourishing. Both indices have been validated in multiple languages and are widely used in cross-national research (Wȩziak-Białowolska et al., 2019a), including the Global Flourishing Study (VanderWeele et al., 2025). The present study used the Korean version of the SFI, following a standardized forward–backward translation protocol to ensure conceptual equivalence with the original instruments.

**Table 1:** The Flourish Index (FI) and Secure Flourish Index (SFI) (VanderWeele, 2017).

Measure		Domain	Code	Questions and response options
SFI	FI	Happiness and life satisfaction	HS1	Overall, how satisfied are you with life these days?
				*0 = Not satisfied at all, 10 = Completely satisfied*
			HS2	In general, how happy or unhappy do you usually feel?
				*0 = Extremely unhappy, 10 = Extremely happy*
		Physical and mental health	H1	In general, how would you rate your physical health?
				*0 = Poor, 10 = Excellent*
			H2	How would you rate your overall mental health?
				*0 = Poor, 10 = Excellent*
		Meaning and purpose	MN1	Overall, to what extent do you feel the things you do in your life are worthwhile?
				*0 = Not at all worthwhile, 10 = Completely worthwhile*
			MN2	I understand my purpose in life.
				*0 = Strongly disagree, 10 = Strongly agree*
		Character and virtue	CV1	I always act to promote good in all circumstances, even in difficult and challenging situations.
				*0 = Not true, 10 = Completely true*
			CV2	I am always able to give up some happiness now for greater happiness later.
				*0 = Not true, 10 = Completely true*
		Close social relationships	CR1	I am content with my friendships and relationships.
				*0 = Strongly disagree, 10 = Strongly agree*
			CR2	My relationships are as satisfying as I would want them to be.
				*0 = Strongly disagree, 10 = Strongly agree*
	Financial and material stability		FS1	How often do you worry about being able to meet regular monthly living expenses?
				*0 = All the time, 10 = Never*
			FS2	How often do you worry about safety, food, or housing?
				*0 = All the time, 10 = Never*

#### 2.1.2 Translation process

The Korean version of the SFI was developed using the translation, review, adjudication, pretesting, documentation (TRAPD) approach (Supplementary Figure S1), a team-based method designed to detect and correct errors and enhance translation quality (Walde and Völlm, 2023). In the translation phase, two bilingual translators with at least a bachelor’s degree in public health or psychology and demonstrated domain expertise independently translated the original English items into Korean. In the review phase, two independent back-translators, each holding a master’s degree or higher in public health, translated the Korean versions back into English without prior exposure to the original items. In the adjudication phase, a five-member panel consisting of the two translators, two back-translators, and a moderator reviewed all versions, resolved discrepancies, and finalized the wording. During the pretesting phase, a pilot test was conducted with 20 participants to identify potential misunderstandings and refine wording for clarity. Finally, in the documentation phase, all meeting records, decision logs, and successive translation versions were archived to ensure full transparency of the adaptation process.

### 2.2 Participants and data collection

Data were collected through an online survey using the computer-assisted web interviewing method between January 30 and February 8, 2024. The target population consisted of Korean adults aged 19–64 years. Participants were recruited from a pre-existing panel of approximately 880,000 members maintained by a professional survey company. Simple random sampling was applied, with quotas set to match the age, sex, and regional distributions of the general population. Among the respondents, 48.8% were females, 37.1% were married, and 75.6% had attained at least a high school education (Supplementary Table S1). Of the 1,668 randomly invited panel members, 1,217 completed the survey, yielding a response rate of 72.9%. To minimize missing data, logic checks were incorporated into the online format, including requiring completion of each item before proceeding and triggering of a pop-up reminder when an item was skipped.

### 2.3 Statistical procedure

#### 2.3.1 Analytical strategies

The psychometric properties of the FI and SFI were evaluated using a combined approach that integrated CTT and IRT. This dual-method strategy enabled a more comprehensive validation by assessing both overall measurement quality and item-level performance. Prior studies have demonstrated that CTT and IRT offer complementary forms of evidence: CTT evaluates reliability and structural validity at the scale level, whereas IRT provides fine-grained information on item characteristics (Embretson and Reise, 2000). Such an integrated approach is particularly valuable for examining how cultural factors shape response patterns and item behavior. For instance, the psychometric evaluation of the Chinese version of the Decision Regret Scale employed both CTT and IRT to assess item contribution, evaluate category functioning, and detect potential differential item functioning (DIF) across subgroups, demonstrating the diagnostic advantages of a multi-method framework in non-Western cultural contexts (Xu et al., 2020).

In this study, we evaluated reliability and construct validity within the CTT framework, including an examination of the relationships between individual items and the overall construct. The IRT framework, specifically the graded response model, was used to investigate detailed item characteristics, such as discrimination, difficulty parameters, and response-category functioning, based on respondents’ latent trait levels. Measurement invariance testing was conducted to examine the stability of the instruments across groups, while DIF analyses were performed to identify items that functioned differently according to key sociodemographic variables, including age, sex, and educational attainment. By combining these approaches, the analyses captured both the general reliability and validity of the scales as well as the nuanced functioning of individual items across diverse population subgroups.

#### 2.3.2 CTT analyses: reliability and construct validity

Within the CTT framework, descriptive statistics (mean, standard deviation, skewness, and kurtosis) were computed for each item to examine response distributions. Reliability and internal consistency were assessed using Cronbach’s alpha and Pearson correlation coefficients between items and domain scores. Construct validity was examined through exploratory factor analysis (EFA) and confirmatory factor analysis (CFA) using the *psych* package for reliability and exploratory analyses (Revelle, 2025); and the *lavaan* package for structural equation modeling (Rosseel, 2012). EFA was conducted using principal axis factoring with oblique (“oblimin”) rotation, which allows correlation among factors (Harman, 1960; Reise et al., 2010). Adequacy for EFA was supported by a Kaiser–Meyer–Olkin statistic of 0.88 for the FI and 0.86 for the SFI, along with significant chi-square values from Bartlett’s test of sphericity (FI: *χ*^2^ = 8673.18, *p* < 0.001; SFI: *χ*^2^ = 9748.43, *p* < 0.001).

In CFA, three alternative models were tested for both the FI and SFI: (a) a unidimensional model that assumed that all items loaded on a single latent factor representing the overall construct (Reise et al., 1993); (b) a correlated traits model that specified multiple domain factors that are conceptually distinct but allowed to correlate (Marsh, 1989); and (c) a hierarchical (second-order) model, in which domain factors were modeled as first-order constructs loading onto a higher-order general factor representing the overarching construct (Chen et al., 2006). Model fit was evaluated using the comparative fit index (CFI), Tucker–Lewis Index (TLI), root mean square error of approximation (RMSEA), and standardized root mean square residual (SRMR), and fit indices were compared to determine the best-fitting model. No residual covariances were permitted in the CFA models to preserve the structural purity of the domain-based measurement framework and avoid overfitting to sample-specific item dependencies. The *semPlot* package for graphical representations (Epskamp, 2022).

Convergent validity was assessed by examining correlations with existing measures of physical and mental health status (i.e., representing good health; multimorbidity, depression, and anxiety), and freedom to determine how to live. Multimorbidity was measured as the self-reported number of physician-diagnosed chronic conditions affecting major systems (cardiovascular, cerebrovascular, musculoskeletal, and respiratory), based on measures used in nationally representative Korean surveys (Kang et al., 2015; Lee et al., 2022). Depression was assessed using the Korean version of the Patient Health Questionnaire-9 (PHQ-9), a validated nine-item scale that evaluates depressive symptoms based on DSM-IV criteria using a 4-point Likert scale (0 = not at all to 3 = nearly every day) (Han et al., 2008; Kroenke et al., 2001). Anxiety was measured using the Korean version of the Generalized Anxiety Disorder-2 (GAD-2), a brief two-item screening tool for generalized anxiety, also using a 4-point Likert scale (Ahn et al., 2019; Plummer et al., 2016). Complementary analyses examined the convergent validity of the SFI with external financial indicators, including perceived freedom to determine one’s life course, self-reported economic status at 12 years, current monthly personal/household income, and the perceived appropriate amount of average monthly living expenses. These analyses were conducted to evaluate the incremental validity of the financial and material stability (D6).

Discriminant validity was evaluated by correlating flourishing scores with self-reported health behaviors: nicotine use, alcohol consumption, and physical activity. Nicotine use was measured by the frequency of current use of conventional cigarettes and heated tobacco products (0 = none, 3 = daily). Alcohol consumption was assessed using the first item of the Korean version of the Alcohol Use Disorders Identification Test (AUDIT-K), which captures drinking frequency (Kim et al., 2008). Physical activity was measured by the number of days per week engaging in moderate-intensity activity. Convergent and discriminant validity were assessed using Pearson correlations. Statistical significance was set at *p* < 0.5.

#### 2.3.3 IRT analyses: item characteristics

The IRT analyses were restricted to the FI, excluding the financial and material stability (D6) domain. This decision was guided by both theoretical and empirical distinctiveness. Conceptually, the Human Flourishing framework posits financial and material stability (D6) as an enabling condition—a means to support and sustain flourishing (VanderWeele, 2017). Empirically, CTT analyses indicated that the financial and material stability (D6) shared limited common variance with the core domains, functioning as a distinct auxiliary factor. In IRT framework, including a conceptually and statistically distinct dimension alongside a cohesive set of core traits can obscure the precision of the parameter estimates. Consequently, the IRT calibration focused exclusively on the FI core domains to ensure construct coherence.

Prior to fitting IRT models, the three fundamental assumptions of IRT were evaluated: unidimensionality, local independence, and monotonicity. Unidimensionality assumes that item responses are primarily explained by a single dominant latent trait; this was assessed by fitting a one-factor CFA model and examining overall model fit. Local independence assumes that, after accounting for the latent trait, item responses are statistically independent; this was evaluated following the estimation of a unidimensional GRM by inspecting residual correlations, with Yen’s Q3 values greater than 0.30 indicating serious violations (Christensen et al., 2017). Monotonicity assumes that the likelihood of endorsing higher response options increases with higher levels of the latent trait (Hemker et al., 1996); this was examined using Mokken scale analysis implemented in the *mokken* package in R (van der Ark, 2007).

A graded response model (GRM) using marginal maximum likelihood estimation was used to analyze the polytomous item responses of the FI. Both unidimensional and multidimensional GRMs were used to estimate item parameters, including discrimination and threshold parameters. Item characteristic curves and item information curves were inspected to evaluate item performance and the information contributed across the latent trait continuum. Unlike the unidimensional GRM, which assumes a single latent continuum, the multidimensional GRM simultaneously estimates multiple correlated latent traits corresponding to the instrument’s domains (Paek and Cole, 2019). This approach enables a more precise evaluation of domain-specific discrimination and threshold parameters, while also identifying cross-domain contributions. As a result, it provides richer insights into how each domain uniquely contributes to the overarching flourishing construct. In the multidimensional GRM, correlations among the five domain-specific latent factors (happiness, health, meaning, character, and relationships) were freely estimated to reflect their conceptual interrelatedness. All IRT analyses were conducted using the *mirt* package (Chalmers, 2012) and visualized with *ggmirt* (Masur, 2025).

#### 2.3.4 Measurement invariance and DIF

Measurement invariance across key sociodemographic variables (sex, age, marital status, education, and religion) was evaluated using multi-group CFA within the CTT framework. A sequential testing procedure was applied to assess: configural invariance, testing whether the same factor structure held across groups without parameter constraints; metric invariance, imposing equality constraints on factor loadings across groups; and scalar invariance, further constraining item intercepts to be equal. Invariance was evaluated using likelihood ratio tests (*p* < 0.05), supplemented by changes in CFI (ΔCFI ≤ 0.01) and RMSEA (ΔRMSEA ≤ 0.015) as recommended cutoffs (Putnick and Bornstein, 2016).

The DIF occurs when individuals with equivalent latent trait levels exhibit differing probabilities of endorsing item responses across groups (e.g., by age, sex, or education), indicating potential measurement bias. DIF was assessed using the iterative hybrid ordinal logistic regression method implemented in the *lordif* package in R (Choi et al., 2011). For each item, nested ordinal logistic regression models were fitted, sequentially adding the grouping variable and its interaction with the latent trait estimate (*θ*) to detect uniform and non-uniform DIF. McFadden’s pseudo-*R*^2^ (Δ*R*^2^) was used as an effect size measure, with values ≥ 0.02 considered indicative of meaningful DIF, while statistical significance was determined using chi-square difference tests (*p* < 0.01). Items meeting both criteria, i.e., statistical significance and effect size above the threshold, were flagged for potential DIF. All statistical analyses were conducted in R, version 4.5.1 (R Core Team, 2025).

## 3 Results

### 3.1 Descriptive statistics, reliability, and correlations

Table 2 presents descriptive statistics, correlations, and reliability estimates for the items of the FI and SFI. Within the character and virtue (D4) domain, two items (CV1: promoting good; CV2: delayed gratification) exhibited high mean scores (mean [SD]: CV1 = 6.99 [1.82]; CV2 = 6.54 [1.99]). In contrast, items in the financial and material stability (D6) domain (FS1: worry about monthly living expenses; FS2: worry about safety, food, or housing) demonstrated lower mean scores (mean [SD]: FS1 = 4.19 [2.51]; FS2 = 4.48 [2.50]). An analysis of univariate skewness and kurtosis showed that all items fell within acceptable limits (±2), consistent with assumptions of univariate normality (Kim, 2013). Most items displayed negative skewness, implying distributions with longer right tails, except for FS1 and FS2.

**Table 2:** Descriptive statistics and reliability for the items of the Flourish Index (FI) and the Secure Flourish Index (SFI) (*N* = 1,217).

Domain	Item (code)	Mean	SD	Skewness	Kurtosis	Corrected item-total correlations	Cronbach’s α if item deleted
D1. Happiness and life satisfaction	HS1	5.78	2.15	−0.59	−0.05	0.76	0.86
	HS2	5.90	2.02	−0.55	0.12	0.79	0.86
D2. Physical and mental health	H1	5.58	1.98	−0.24	−0.15	0.63	0.87
	H2	6.00	2.21	−0.45	−0.26	0.77	0.86
D3. Meaning and purpose	MN1	6.26	2.10	−0.64	0.39	0.75	0.86
	MN2	6.29	2.24	−0.58	0.04	0.73	0.87
D4. Character and virtue	CV1	6.99	1.82	−0.66	0.70	0.50	0.88
	CV2	6.54	1.99	−0.60	0.43	0.35	0.89
D5. Close social relationship	CR1	6.43	2.02	−0.59	0.39	0.62	0.87
	CR2	6.13	2.08	−0.42	−0.01	0.65	0.87
D6. Financial and material stability	FS1	4.19	2.51	0.23	−0.53	0.26	0.90
	FS2	4.48	2.50	0.15	−0.63	0.33	0.89

SD, standard deviation.

The overall internal consistency of the total scale, as measured by Cronbach’s alpha, was 0.88 (95% CI: 0.87–0.89), indicating good reliability. However, items within the character and virtue (D4) domain (CV1 = 0.50; CV2 = 0.35) and the financial and material stability (D6) domain (FS1 = 0.26; FS2 = 0.33) demonstrated weaker consistency with the total scale. Excluding these items slightly improved the overall internal consistency of the FI. Correlation patterns are presented in Supplementary Figure S2 (item-level heatmap) and Supplementary Table S2 (subdomain intercorrelation matrix). The subdomains of the FI and SFI demonstrated positive correlations with each other, ranging from 0.407 to 0.740. Stronger correlations were observed among the domains of happiness and life satisfaction (D1), physical and mental health (D2), and meaning and purpose (D3), with coefficients exceeding 0.70. Within-domain consistency was also high, with intraclass correlation coefficients of 0.86 for D1 and 0.87 for D3.

### 3.2 Dimensionality and factor structure

To evaluate the dimensional structure of the FI and SFI, factor analyses were performed. Parallel analyses based on both principal components and principal axis factoring were first conducted to estimate the optimal number of factors. Results indicated five factors and one component for the FI, and six factors and two components for the SFI (Supplementary Figure S3). EFA was then conducted to examine the detailed factor structure and guide subsequent confirmatory model specifications. Factor loadings and model fit indices for the FI are presented in Table 3, and those for the SFI are presented in Table 4. The results support the hypothesized theoretical factor structure of both instruments. The best-fitting models were a five-factor solution for the FI (TLI = 0.975, RMSEA = 0.062) and a six-factor solution for the SFI (TLI = 0.981, RMSEA = 0.048), with model fit indices indicating strong fits. Factor loadings ranged from 0.514 to 0.935.

**Table 3:** Factor loadings and the model fit indices for exploratory factor analysis of the Flourish Index.

Item (code)	One-factor solution	Two-factor solution		Three-factor solution			Four-factor solution				Five-factor solution				
	F1	F1	F2	F1	F2	F3	F1	F2	F3	F4	F1	F2	F3	F4	F5
HS1	0.82	0.88	–	0.95	–	–	0.94	–	–	–	–	0.89	–	–	–
HS2	0.85	0.91	–	0.94	–	–	0.82	–	–	–	–	0.90	–	–	–
H1	0.68	0.67	–	0.59	–	–	–	–	0.79	–	–	–	–	0.79	–
H2	0.83	0.83	–	0.72	–	–	–	–	0.81	–	–	–	–	0.76	–
MN1	0.82	0.82	–	0.67	–	–	0.43	–	–	–	–	–	0.83		–
MN2	0.80	0.76	–	0.57	–	–	–	–	–	0.50	–	–	0.85	–	–
CV1	0.55	0.35	–	–	–	0.41	–	–	–	0.49	–	–	–	–	0.55
CV2	0.41	–	–	–	–	0.47	–	–	–	0.55	–	–	–	–	0.51
CR1	0.68	–	0.93	–	0.96	–	–	0.95	–	–	0.93	–	–	–	–
CR2	0.71	–	0.92	–	0.93	–	–	0.90	–	–	0.92	–	–	–	–
Model fit indices															
TLI	0.71	0.872		0.926			0.932				0.975				
RMSEA	0.216	0.142		0.108			0.104				0.062				
BIC	1767.4	480.68		145.49			76.44				−7.01				

HS1, Life satisfaction; HS2, Happiness; H1, Physical health; H2, Mental health; MN1, Meaningfulness; MN2, Purposefulness; CV1, Promoting good; CV2, Delayed gratification; CR1, Close social relationship; CR2, Satisfaction on close relationship; FS1, Worry about monthly living expenses; FS2, Worry about safety, food, or housing. TLI, Tucker-Lewis Index; RMSEA, Root-mean-square error of approximation; BIC, Bayesian information criterion.

**Table 4:** Factor loadings and model fit indices for exploratory factor analysis of Secure Flourish Index.

Item	One-factor solution	Two-factor solution		Three-factor solution			Four-factor solution				Five-factor solution					Six-factor solution					
	F1	F1	F2	F1	F2	F3	F1	F2	F3	F4	F1	F2	F3	F4	F5	F1	F2	F3	F4	F5	F6
HS1	0.82	0.79	–	0.86	–	–	0.95	–	–	–	0.90	–	–	–	–	–	0.88	–	–	–	–
HS2	0.85	0.82	–	0.89	–	–	094	–	–	–	0.78	–	–	–	–	–	0.90	–	–	–	–
H1	0.68	0.66	–	0.66	–	–	0.56	–	–	–	–	–	0.78	–	–	–	–	–	–	0.77	–
H2	0.83	0.82	–	0.83	–	–	0.68	–	–	–	–	–	0.76	–	–	–	–	–	–	0.69	–
MN1	0.82	0.82	–	0.84	–	–	0.63	–	–	–	–	–	–	–	0.41	–	–	0.83	–	–	–
MN2	0.80	0.81	–	0.79	–	–	0.51	–	–	–	–	–	–	–	0.56	–	–	0.85	–	–	–
CV1	0.54	0.58	–	0.39	–	–	–	–	–	0.42	–	–	–	—	0.49	–	–	–	–	–	0.57
CV2	0.40	0.45	–	0.32	–	–	–	–	–	0.48	–	–	–	–	0.53	–	–	–	–	–	0.51
CR1	0.68	0.71	–	–	0.93	–	–	0.95	–	–	–	0.95	–	–	–	0.93	–	–	–	–	–
CR2	0.70	0.73	–	–	0.93	–	–	0.93	–	–	–	0.91	–	–	–	0.92	–	–	–	–	–
FS1	0.21		0.86	–	–	0.86	–	–	0.86	–	–	–	–	0.86	–	–	–	–	0.86	–	–
FS2	0.27		0.82	–	–	0.84	–	–	0.86	–	–	–	–	0.86	–	–	–	–	0.86	–	–
Model fit indices																					
TLI	0.633	0.69		0.872			0.93				0.942					0.981					
RMSEA	0.21	0.193		0.124			0.092				0.083					0.048					
BIC	2574.75	1688.17		417.22			98.47				37.66					−30.21					

HS1, Life satisfaction; HS2, Happiness; H1, Physical health; H2, Mental health; MN1, Meaningfulness; MN2, Purposefulness; CV1, Promoting good; CV2, Delayed gratification; CR1, Close social relationship; CR2, Satisfaction on close relationship; FS1, Worry about monthly living expenses; FS2, Worry about safety, food, or housing. TLI, Tucker-Lewis Index; RMSEA, Root-mean-square error of approximation; BIC, Bayesian information criterion.

**Figure 1:**
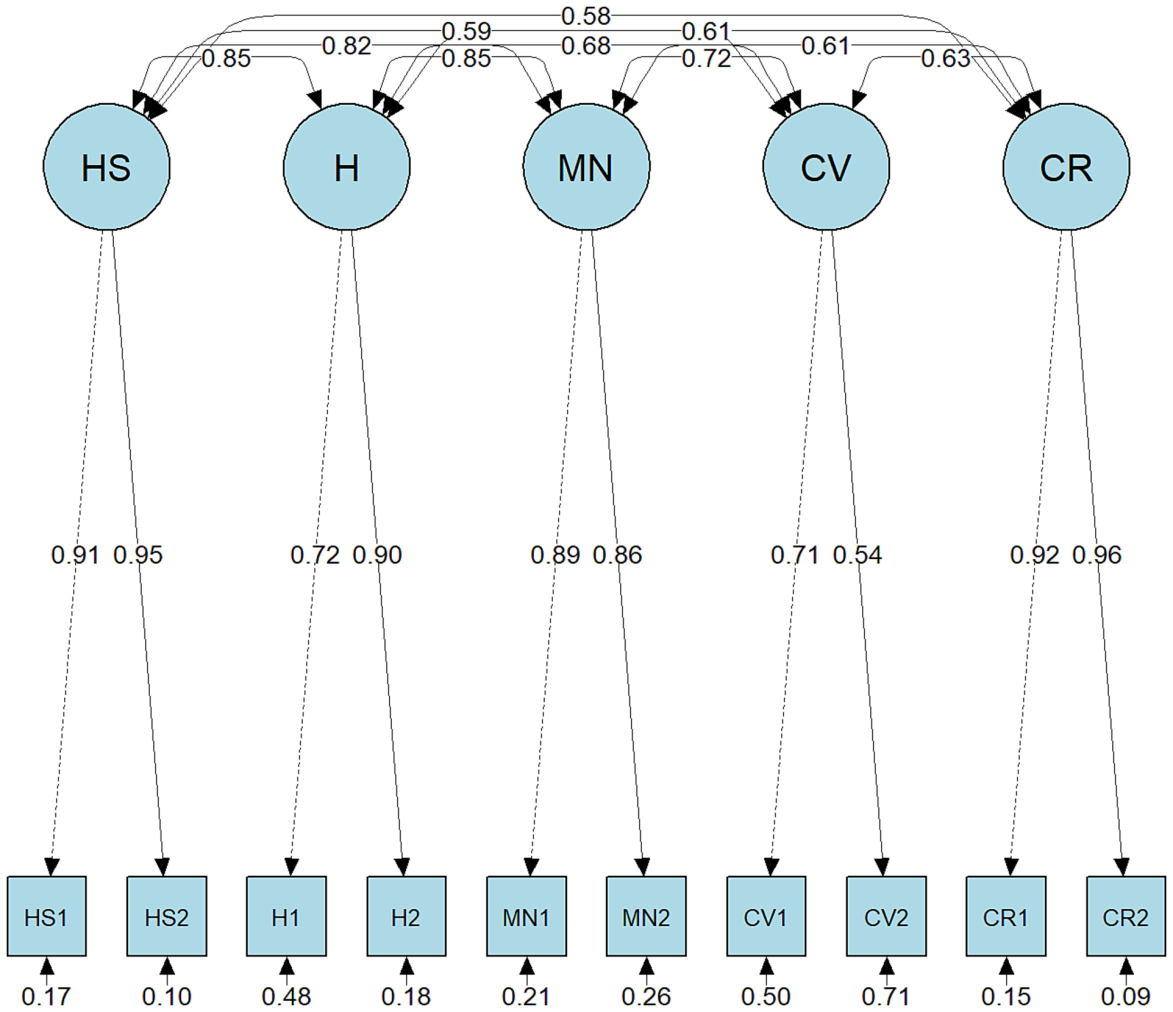
Confirmatory factor analysis results of the Flourish Index. HS, Happiness and life satisfaction; H, Physical and mental health; MN, Meaning and purpose; CV, Character and virtue; CR, Close social relationships.

**Figure 2:**
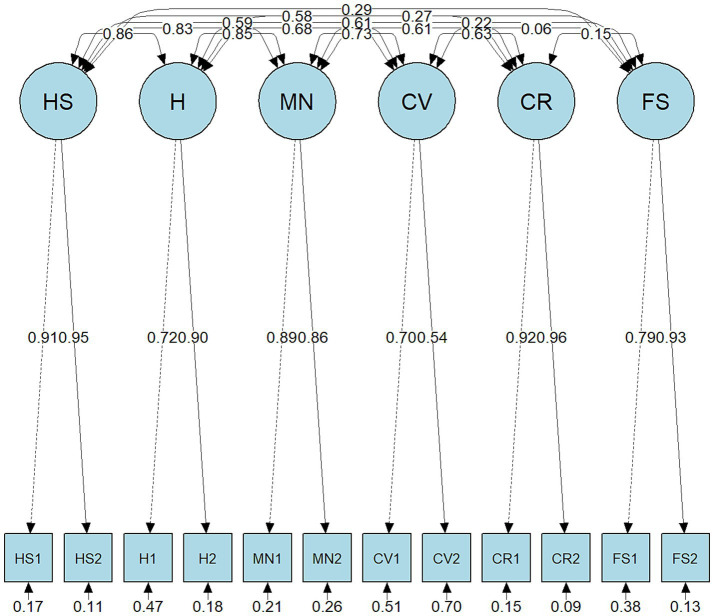
Confirmatory factor analysis results of the Secure Flourish Index. HS, Happiness and life satisfaction; H, Physical and mental health; MN, Meaning and purpose; CV, Character and virtue; CR, Close social relationships; FS, Financial and material stability.

The CFA was subsequently performed to compare unidimensional, correlated traits, and second-order models for both FI and SFI. As shown in Table 5, the correlated traits model demonstrated superior fit for the FI (CFI = 0.995, TLI = 0.991, RMSEA = 0.038, SRMR = 0.014) as well as the SFI (CFI = 0.994, TLI = 0.990, RMSEA = 0.035, SRMR = 0.019), with lower AIC, and BIC compared to alternative specifications. Accordingly, the correlated-traits model was selected as the preferred structure for subsequent analyses. The correlated traits model path diagrams are presented in Figure 1 (FI) and Figure 2 (SFI). Factor loadings for the FI (five-factor model) and SFI (six-factor model) ranged from 0.54 to 0.96 (Table 6), indicating that all items demonstrated adequate loadings on their respective factors.

**Table 5:** Model fit indices for the confirmatory factor analysis (CFA).

Fit index	Uni-dimensional	Correlated-traits	Second-order
A. Flourish index (FI)			
CFI	0.777	**0.995**	0.988
TLI	0.713	**0.991**	0.982
RMSEA	0.213	**0.038**	0.053
SRMR	0.080	**0.014**	0.030
AIC	45413.65	**43531.31**	43586.84
BIC	45515.74	**43684.44**	43714.44
B. Secure Flourish Index (SFI)			
CFI	0.705	**0.994**	0.986
TLI	0.639	**0.990**	0.980
RMSEA	0.209	**0.035**	0.049
SRMR	0.106	**0.019**	0.037
AIC	56663.72	**53867.65**	53942.36
BIC	56786.22	**54066.71**	54095.49

CFI, Comparative Fit Index; TLI, Tucker–Lewis Index; RMSEA, Root-mean-square error of approximation; SRMR, Standardized root mean square residual; AIC, Akaike information criterion; BIC, Bayesian information criterion. For each fit index, the best-fitting values across the three CFA models are highlighted in bold; the correlated-traits model consistently demonstrated the most favorable fit for both the FI and SFI.

**Table 6:** Confirmatory factor analysis standardized factor loadings for correlated traits model.

Item (code)	Flourish index					Secure flourish index					
	HS	H	MN	CV	CR	HS	H	MN	CV	CR	FS
HS1	0.913	–	–	–	–	0.917	–	–	–	–	–
HS2	0.949	–	–	–	–	0.948	–	–	–	–	–
H1	–	0.754	–	–	–	–	0.761	–	–	–	–
H2	–	0.905	–	–	–	–	0.898	–	–	–	–
MN1	–	–	0.895	–	–	–	–	0.894	–	–	–
MN2	–	–	0.873	–	–	–	–	0.872	–	–	–
CV1	–	–	–	0.713	–	–	–	–	0.693	–	–
CV2	–	–	–	0.538	–	–	–	–	0.559	–	–
CR1	–	–	–	–	0.922	–	–	–	–	0.919	–
CR2	–	–	–	–	0.966	–	–	–	–	0.971	–
FS1	–	–	–	–	–	–	–	–	–	–	0.783
FS2	–	–	–	–	–	–	–	–	–	–	0.957

HS, Happiness and life satisfaction; H, Physical and mental health; MN, Meaning and purpose; CV, Character and virtue; CR, Close social relationships; FS, Financial and material stability; HS1, Life satisfaction; HS2, Happiness; H1, Physical health; H2, Mental health; MN1, Meaningfulness; MN2, Purposefulness; CV1, Promoting good; CV2, Delayed gratification; CR1, Close social relationship; CR2, Satisfaction on close relationship; FS1, Worry about monthly living expenses; FS2, Worry about safety, food, or housing.

### 3.3 Convergent and discriminant validity

Correlations with external variables used to assess convergent and discriminant validity are presented in Table 7. To assess convergent validity, we examined the associations between flourishing scores, both the full indices (the FI and SFI) and each individual domain, and theoretically related health-status indicators, including chronic conditions, depression, and anxiety. For discriminant validity, we compared flourishing scores with theoretically distinct constructs, specifically health behaviors such as smoking, alcohol consumption, and physical activity. Strong correlations with health status measures were interpreted as evidence of convergent validity, whereas weak correlations with health behaviors were taken as indicative of discriminant validity. The results show that flourishing scores, particularly the FI and SFI, were strongly associated with mental health indicators, such as depression and anxiety. In contrast, their associations with smoking, alcohol use, and physical activity were weak, thereby providing evidence for both convergent and discriminant validity.

**Table 7:** Correlations of the Flourish Index and Secure Flourish Index with external variables.

Measure	FI	SFI	HS (D1)	H (D2)	MN (D3)	CV (D4)	CR (D5)	FS (D6)
Convergent validity (health status, and autonomy)								
Multi-morbidity	−0.113^*^	−0.128^***^	−0.072	−0.160^***^	−0.078	−0.071	−0.077	−0.091
Depression [PHQ-9]	−0.585^***^	−0.616^***^	−0.549^***^	−0.560^***^	−0.525^***^	−0.284^***^	−0.418^***^	−0.309^***^
Anxiety [GAD-2]	−0.461^***^	−0.502^***^	−0.447^***^	−0.428^***^	−0.416^***^	−0.189^***^	−0.352^***^	−0.306^***^
Discriminant validity (health behaviors)	–	–	–	–	–	–	–	–
Nicotine use status	−0.092^**^	−0.121^***^	−0.109^***^	−0.072^*^	−0.107^***^	−0.055	−0.026	−0.026^***^
Number of days drinking alcohol	0.015	0.027	0.044	−0.010	0.030	0.020	−0.025	−0.025
Number of days practicing intermediate-level physical activity	0.162^***^	0.174^***^	0.118^***^	0.178^***^	0.129^***^	0.087^***^	0.139^***^	0.139^***^

*p < 0.05, **p < 0.01, ***p < 0.001.

FI, Flourish Index; SFI, Secure Flourish Index; HS; happiness and life satisfaction; H, physical and mental health; MN, meaning and purpose; CV, character and virtue; CR, close social relationships; FS, financial and material stability.

Additional analyses examined the convergent validity of the Financial and Material Stability domain (D6) using external financial indicators (see Supplementary Table S3). Results showed that D6 was positively correlated with economic status variables. Accordingly, the SFI demonstrated stronger associations than the FI with perceived economic indicators, including self-rated economic status at age 12, current monthly personal or household income, and the perceived appropriate amount of monthly living expenses. However, the SFI did not show stronger convergence than the FI with perceived freedom to determine one’s life course.

### 3.4 Measurement invariance

Measurement invariance was evaluated across sex, age, marital status, education, and religion using a sequence of increasingly constrained models: configural, metric, and scalar invariance (Supplementary Tables S4, S5). The configural, metric, and scalar models for both the FI and SFI demonstrated acceptable fits. No substantial changes in goodness-of-fit indices, such as the CFI and TLI, were observed between the configural and metric models across groups for either the FI or the SFI. Similarly, changes in RMSEA values remained within the recommended threshold of ≤0.010 (Cheung and Rensvold, 2002). These findings support the equivalence of factor loadings across groups. In contrast, significant differences were observed between the metric and scalar models for sex, age, and marital status groups in the FI, and for sex, age, marital status, and education groups in the SFI. These results indicate that intercept-level measurement invariance was not supported across sex, age, or marital status for the FI, nor across sex, age, marital status, or education for the SFI.

### 3.5 Model and item-level analysis: IRT application

#### 3.5.1 Assumptions

Three fundamental assumptions were evaluated: unidimensionality, local independence, and monotonicity (Supplementary Tables S6, S7 and Supplementary Figure S4). First, to assess the assumption of unidimensionality, a one-factor CFA was performed. The fit indices from the one-factor CFA did not meet the thresholds for excellent fit (CFI = 0.775, TLI = 0.711, RMSEA = 0.214). Local independence was examined by analyzing residual correlations in the unidimensional GRM. Residual correlations below |0.20| were considered evidence that the assumption was met, whereas values exceeding |0.30| were interpreted as clear violations (Christensen et al., 2017). The residual correlation between the two items in the character and virtue domain was particularly high (*ρ* = 0.785). Additionally, correlations between the two items in the happiness and life satisfaction domain (*ρ* = 0.368), as well as between HS2 (happiness) and MN2 (purposefulness) (*ρ* = 0.339), exceeded the threshold, implying multidimensionality across subscales (Supplementary Table S5). Therefore, a bifactor GRM analysis was conducted, using the dimensional structure identified in the EFA as the basis for model specification. Lastly, most items demonstrated acceptable monotonicity, with Loevinger’s *H* coefficients exceeding 0.5 (Supplementary Table S6 and Supplementary Figure S4). However, CV1 (promoting good) and CV2 (delayed gratification) showed minor deviations from this assumption, with negligible violation rates.

#### 3.5.2 Unidimensional GRM

Results of the IRT analysis using the GRM are presented in Table 8. The overall model fit did not meet the recommended thresholds (AIC = 43,641.65, BIC = 44,203.0, CFI = 0.893, TLI = 0.862, RMSEA = 0.197). Each item was evaluated based on its discrimination parameter (Alpha), category threshold parameters (Beta, B1 through B10), and fit statistics, including information-weighted fit (infit) and outlier-sensitive fit (outfit) mean-square values.

**Table 8:** Parameter estimates for the unidimensional graded response model of the Flourish Index.

Item	Discrimination (slope)	Difficulty (beta)										Infit	Outfit
	General	B1	B2	B3	B4	B5	B6	B7	B8	B9	B10		
HS1	3.247	−2.39	−2.02	−1.60	−1.06	−0.82	−0.26	0.19	0.83	1.71	2.47	0.92	0.997
HS2	3.612	−2.48	−2.17	−1.77	−1.22	−0.88	−0.27	0.16	0.79	1.67	2.38	0.87	0.88
H1	1.914	−3.28	−2.67	−2.02	−1.37	−0.82	−0.03	0.48	1.24	2.16	2.94	0.998	1.02
H2	3.006	−2.62	−2.17	−1.74	−1.20	−0.84	−0.27	0.10	0.65	1.40	2.16	0.89	0.89
MN1	2.914	−2.62	−2.27	−1.93	−1.44	−1.13	−0.47	−0.01	0.60	1.46	2.00	0.93	0.91
MN2	2.622	−2.55	−2.33	−1.80	−1.38	−1.06	−0.45	−0.04	0.55	1.28	1.91	0.95	0.98
CV1	1.228	−4.91	−4.56	−3.78	−2.99	−2.43	−1.41	−0.59	0.35	1.40	2.67	0.99	0.95
CV2	0.893	−5.52	−5.00	−4.19	−3.16	−2.28	−1.23	−0.34	0.85	2.27	3.57	1.03	1.05
CR1	1.568	−3.55	−3.19	−2.78	−1.99	−1.56	−0.65	−0.12	0.72	1.59	2.56	0.97	1.02
CR2	1.683	−3.26	−3.06	−2.56	−1.71	−1.14	−0.43	0.14	0.85	1.72	2.60	0.98	1.01

HS1, Life satisfaction; HS2, Happiness; H1, Physical health; H2, Mental health; MN1, Meaningfulness; MN2, Purposefulness; CV1, Promoting good; CV2, Delayed gratification; CR1, Close social relationship; CR2, Satisfaction on close relationship; FS1, Worry about monthly living expenses; FS2, Worry about safety, food, or housing; Infit, Information-weighted fit; Outfit, Outlier-sensitive fit.

The discrimination parameter can theoretically vary from −∞ to +∞; however, in empirical research it typically ranges from 0 to 2.0 (Edelen and Reeve, 2007). Items with higher discrimination values provide more information and are more sensitive to differences in the underlying trait, with coefficients exceeding 1.7 considered highly discriminative (Baker, 2001). The estimated discrimination parameters of the unidimensional GRM ranged from 0.893 to 3.612, with a mean of 2.269, indicating a moderate to high capacity to differentiate respondents along the latent trait continuum. Items in the happiness and life satisfaction, health, and meaning and purpose domains showed higher levels of difficulty, whereas items in the character and virtue domain exhibited lower difficulty. In particular, CV2 (delayed gratification) demonstrated the lowest difficulty, while HS2 (happiness) exhibited the highest. Difficulty threshold parameters (B1 to B10) generally spanned the latent trait scale from approximately −4.91 to 3.57, implying that the items collectively covered a broad range of the construct. Items from character and virtue spanned the widest range of thresholds, whereas items from meaning and purpose covered the narrowest range. The item fit statistics, both infit and outfit, were close to 1 for all items, with no outliers detected.

**Figure 3:**
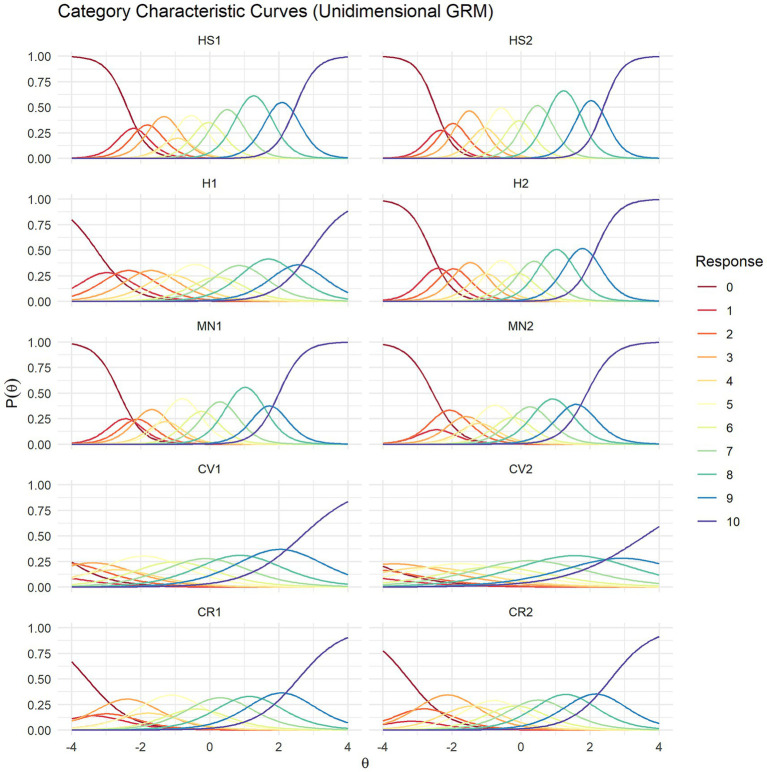
Category characteristic curves of the unidimensional graded response model of the Flourish Index. HS1, Life satisfaction; HS2, Happiness; H1, Physical health; H2, Mental health; MN1, Meaningfulness; MN2, Purposefulness; CV1, Promoting good; CV2, Delayed gratification; CR1, Close social relationship; CR2, Satisfaction with close relationship.

The category characteristic curves of the unidimensional GRM, shown in Figure 3 (boundary characteristic curves are presented in Supplementary Figure S5), describe the transition of responses across categories. HS1 (life satisfaction), HS2 (happiness), H2 (mental health), MN1 (meaningfulness), and MN2 (purposefulness) displayed the expected pattern, with higher levels of the latent trait corresponding to an increased probability of selecting higher response categories. The response probability curves were generally narrow and peaked, indicating that these items effectively discriminated between response categories across different levels of the trait. In contrast, H1 (physical health) exhibited slightly more dispersed response curves, although the overall response pattern remained consistent with expectations. Items CV1 (promoting good), CV2 (delayed gratification), and CR (close social relationships) demonstrated low discrimination. In the character and virtue domain (CV1, CV2), the curves exhibited substantial overlap, with high probabilities for the lower response categories (0, 1, and 2) at low latent trait levels. Furthermore, probabilities for higher response categories were concentrated at relatively low trait levels, reflecting low item difficulty. Items in close social relationships (CR1, CR2) showed flattened and consistently low response probabilities in the higher trait range, particularly reflecting limited discrimination at the lower end.

**Figure 4:**
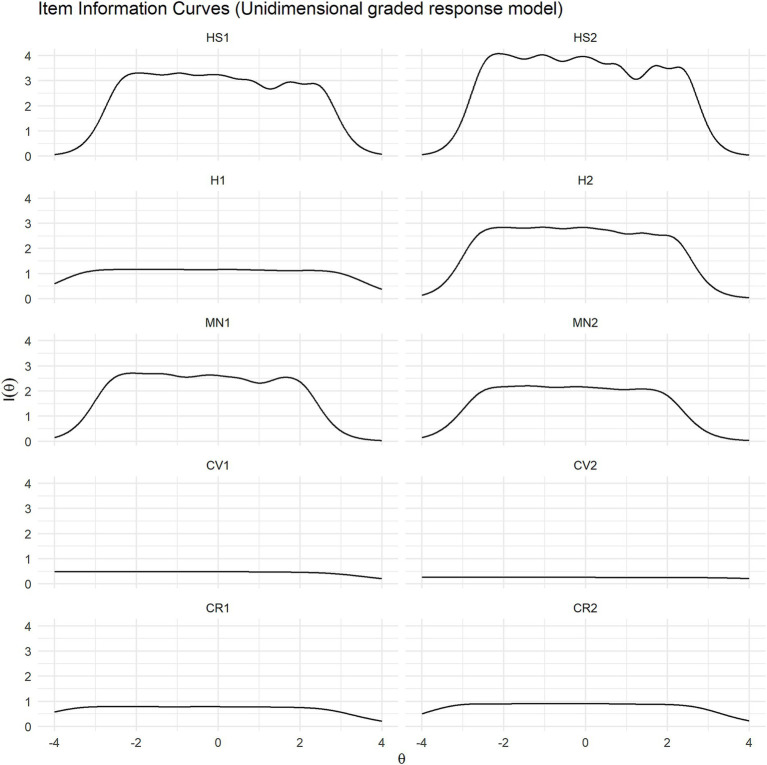
Item information curves of the unidimensional graded response model of the Flourish Index. HS1, Life satisfaction; HS2, Happiness; H1, Physical health; H2, Mental health; MN1, Meaningfulness; MN2, Purposefulness; CV1, Promoting good; CV2, Delayed gratification; CR1, Close social relationship; CR2, Satisfaction with close relationship.

Higher values on the item information characteristic curves indicated greater precision in measuring the latent trait at specific points on the *θ* continuum. The highest levels of information were observed at low to moderate levels of the latent trait, indicating that the items were most precise in measuring respondents with mid-range trait levels (Figure 4). Items in the happiness and life satisfaction domain (HS1, HS2) provided the most information, followed by H2 (mental health), MN1 (meaning), and MN2 (purpose), all of which showed adequate performance (information values ranging from 2 to 3). By contrast, items from the character and virtue (CV1, CV2) and close social relationships (CR1, CR2) domains contributed minimal information.

The unidimensional GRM provided a detailed representation of how each item functioned along the general factor. However, the overall fit indices did not meet recommended thresholds, and several item pairs exhibited residual correlations exceeding acceptable criteria within domains, for example, HS1 (life satisfaction) and HS2 (happiness) (*ρ* = 0.368), and CV1 (promoting good) and CV2 (delayed gratification) (*ρ* = 0.785). These results indicate violations of the local independence assumption and suggested potential multidimensionality. To address this issue, an additional IRT analysis was conducted using a multidimensional GRM.

#### 3.5.3 Multidimensional GRM

The multidimensional GRM captured the five theorized domains of the FI by modeling their unique latent trait characteristics while permitting inter-domain correlations, thereby reflecting both the distinctiveness and the conceptual overlap inherent in the flourishing construct. This approach also addressed the violations of the local independence assumption observed in the unidimensional GRM analysis. The model demonstrated good fit indices, showing marked improvement over the unidimensional GRM (AIC = 41,924.65, BIC = 42,537.14, CFI = 0.986, TLI = 0.992, RMSEA = 0.063). However, the item-level parameters and fit statistics (information-weighted fit and outlier-sensitive fit), presented in Table 9, indicated misfit for several items. Specifically, HS2 (happiness) and the items in the close social relationships domain (CR1 and CR2) exhibited significantly higher discrimination, indicating increased sensitivity to differences in latent traits. Moreover, these items exhibited significantly low infit and outfit mean-square values (both 0.5–0.6), consistent with overfit and indicative of excessively predictable response patterns (Linacre, 2002).

**Table 9:** Parameter estimates for the multidimensional graded response model of the Flourish Index.

Item	Discrimination (slope)					Difficulty (intercept)										Infit	Outfit
	A1	A2	A3	A4	A5	B1	B2	B3	B4	B5	B6	B7	B8	B9	B10		
HS1	4.825	–	–	–	–	−2.128	−1.808	−1.444	−0.958	−0.729	−0.238	0.168	0.757	1.539	2.195	0.768	0.741
HS2	6.363	–	–	–	–	−2.197	−1.911	−1.577	−1.091	−0.787	−0.250	0.135	0.707	1.499	2.111	0.581	0.545
H1	–	2.169	–	–	–	−3.034	−2.484	−1.888	−1.285	−0.774	−0.038	0.432	1.145	1.994	2.710	0.960	0.976
H2	–	3.715	–	–	–	−2.410	−2.008	−1.614	−1.126	−0.786	−0.261	0.088	0.600	1.282	1.975	0.717	0.699
MN1	–	–	3.623	–	–	−2.391	−2.081	−1.776	−1.352	−1.076	−0.462	−0.030	0.533	1.315	1.801	0.807	0.791
MN2	–	–	3.388	–	–	−2.301	−2.109	−1.647	−1.276	−0.995	−0.435	−0.056	0.483	1.133	1.684	0.835	0.855
CV1	–	–	–	1.666	–	−3.954	−3.692	−3.098	−2.479	−2.031	−1.201	−0.519	0.260	1.130	2.147	0.930	0.895
CV2	–	–	–	1.200	–	−4.351	−3.964	−3.342	−2.538	−1.851	−1.024	−0.309	0.653	1.799	2.812	0.992	1.025
CR1	–	–	–	–	5.288	−2.324	−2.118	−1.889	−1.410	−1.121	−0.490	−0.101	0.481	1.076	1.693	0.699	0.681
CR2	–	–	–	–	6.112	−2.205	−2.085	−1.798	−1.252	−0.854	−0.344	0.076	0.582	1.183	1.736	0.575	0.554

HS1, Life satisfaction; HS2, Happiness; H1, Physical health; H2, Mental health; MN1, Meaningfulness; MN2, Purposefulness; CV1, Promoting good; CV2, Delayed gratification; CR1, Close social relationship; CR2, Satisfaction on close relationship; FS1, Worry about monthly living expenses; FS2, Worry about safety, food, or housing; Infit, Information-weighted fit; Outfit, Outlier-sensitive fit.

Further inspection of factor loadings and communality values in Table 10 revealed that these items also displayed exceptionally high communality values, exceeding 0.9, indicating that nearly all their variance was explained by multiple latent traits. Examination of the factor loadings of each item showed that most items loaded strongly on their respective domain factors. The character and virtue domain was an exception, as its items showed moderate to high loadings on the domain factor, but markedly low communality values. In contrast, both HS2 (happiness) and the items in the close social relationships domain (CR1, CR2) exhibited high domain factor loadings along with high communality values. Additionally, an analysis of the response distributions for the happiness and life satisfaction and close social relationships domains revealed highly similar response patterns among item pairs within their respective domains (Supplementary Figure S6).

**Table 10:** Factor loadings and communality value of multidimensional GRM of the FI.

Item	F1	F2	F3	F4	F5	H2 (communality)
HS1	0.943	–	–	–	–	0.889
HS2	0.966	–	–	–	–	0.933
H1	–	0.787	–	–	–	0.619
H2	–	0.909	–	–	–	0.827
MN1	–	–	0.905	–	–	0.819
MN2	–	–	0.894	–	–	0.799
CV1	–	–	–	0.700	–	0.489
CV2	–	–	–	0.576	–	0.332
CR1	–	–	–	–	0.952	0.906
CR2	–	–	–	–	0.963	0.928

HS1, Life satisfaction; HS2, Happiness; H1, Physical health; H2, Mental health; MN1, Meaningfulness; MN2, Purposefulness; CV1, Promoting good; CV2, Delayed gratification; CR1, Close social relationship; CR2, Satisfaction on close relationship; FS1, Worry about monthly living expenses; FS2, Worry about safety, food, or housing.

**Figure 5:**
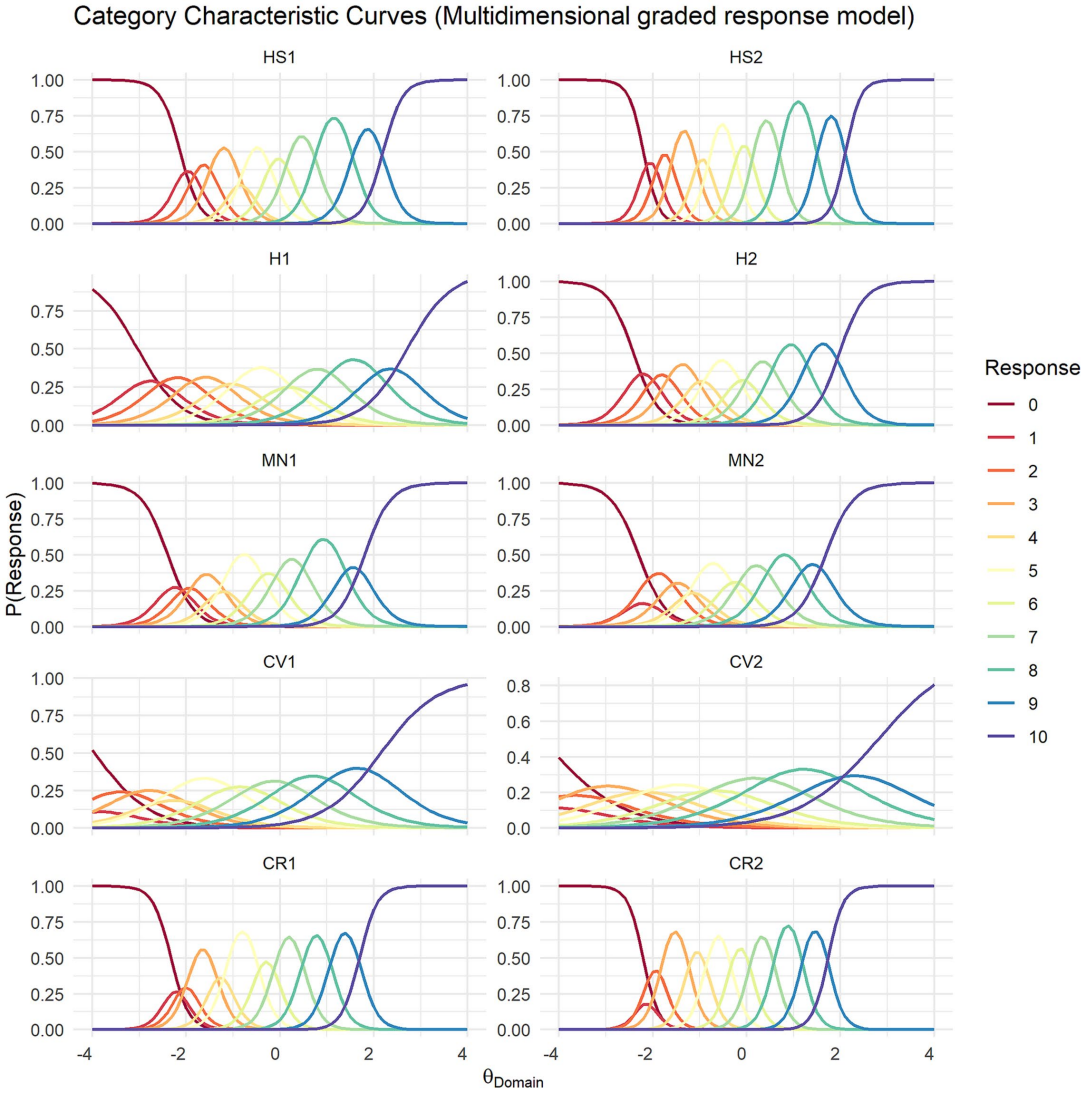
Category characteristic curves of the multidimensional graded response model of the Flourish Index. HS1, Life satisfaction; HS2, Happiness; H1, Physical health; H2, Mental health; MN1, Meaningfulness; MN2, Purposefulness; CV1, Promoting good; CV2, Delayed gratification; CR1, Close social relationship; CR2, Satisfaction with close relationship.

**Figure 6:**
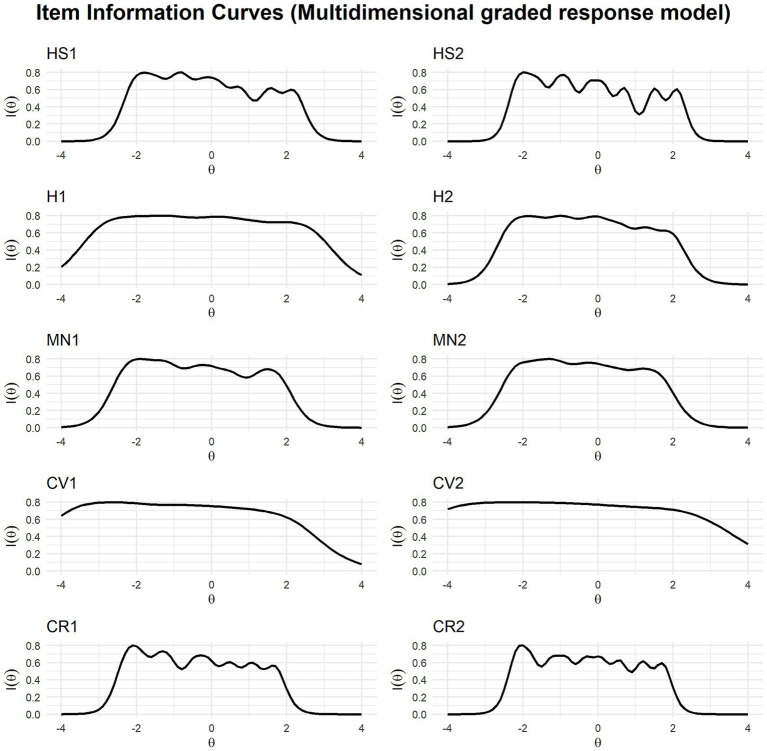
Item information curves of the multidimensional graded response model of the Flourish Index. HS1, Life satisfaction; HS2, Happiness; H1, Physical health; H2, Mental health; MN1, Meaningfulness; MN2, Purposefulness; CV1, Promoting good; CV2, Delayed gratification; CR1, Close social relationship; CR2, Satisfaction with close relationship.

Figure 5 presents the category characteristic curves of the multidimensional GRM. Overall, the curves became narrower and more peaked, while maintaining patterns comparable to those in the unidimensional GRM analysis. However, the two items in the close social relationships domain (CR1, CR2) were exceptions: in the multidimensional GRM, their curves became even narrower and more peaked. Similarly, the item information curves shown in Figure 6 indicated an overall decrease in information under the multidimensional GRM, although the general patterns remained similar. Nevertheless, the two items in the close social relationships domain (CR1, CR2) exhibited more distinct information curves, with relatively more information observed at lower levels of the latent trait.

### 3.6 DIF analysis

To assess measurement invariance across subgroups, a DIF analysis was conducted. DIF tests whether individuals from different groups, matched on the underlying latent trait, have differing probabilities of endorsing item response categories, thereby indicating potential item bias (Zumbo, 1999; Holland and Wainer, 2012). Ten items on the FI were analyzed across sex, age, and marital status groups (Supplementary Table S8 and Supplementary Figures S7, S8). No evidence of statistically significant DIF was found.

## 4 Discussion

Grounded in the World Health Organization’s definition of health, the construct of human flourishing has been conceptualized as an overarching state of “complete well-being” that integrates multiple life domains (VanderWeele, 2017). VanderWeele’s multidimensional framework offers a concise yet comprehensive indicator for monitoring well-being in both research and population health contexts. In this study, we translated and validated the FI and SFI, providing evidence of their psychometric robustness. Furthermore, our findings offer novel insights into how the multidimensional construct of flourishing manifests in the Korean context, as well as highlighting areas for refinement.

The factor structures of both the FI and SFI were consistent with theoretical expectations and empirical evidence from multiple countries, supporting their conceptual and structural validity (VanderWeele, 2017; Wȩziak-Białowolska et al., 2019a; Wȩziak-Białowolska et al., 2019b; Oulevey Bachmann et al., 2023; Sattler et al., 2023). In our data, both the five-factor model of the FI and the six-factor model of the SFI were broadly supported by exploratory and confirmatory factor analyses. The predefined item groupings corresponding to each instrument’s domains, including happiness and life satisfaction, physical and mental health, meaning and purpose, character and virtue, close social relationships, and, in the case of the SFI, financial and material stability, were also confirmed, indicating that the configuration of each scale aligns with the intended conceptual framework (VanderWeele, 2017). In the correlated traits models of both the FI and SFI, all domains were interrelated; however, Character and Virtue (D4) and Financial and Material Stability (D6) showed relatively weak correlations with the overall scale. These findings are consistent with prior theoretical and empirical work regarding the distinct roles of Character and Virtue (D4) and Financial and Material Stability (D6) (VanderWeele, 2017; Węziak-Białowolska et al., 2017). Similar patterns have been reported in validation studies of the FI and SFI translated into other languages, including German, Danish, and French (Oulevey Bachmann et al., 2023; Sattler et al., 2023; Stripp et al., 2024). Character and Virtue showed weaker links with the other domains, possibly because flourishing extends beyond subjective well-being to encompass moral and eudaimonic aspects focused on human potential and ethical conduct (Symons and VanderWeele, 2024; VanderWeele, 2017). In contrast, Financial and Material Stability is theorized as a supportive domain that enables the expression of the five core domains, which may account for its weaker empirical associations (VanderWeele, 2017).

Unlike previous studies applying correlated traits models of the FI and SFI, our findings revealed a distinct pattern of domain interrelationships. In our sample, three domains (happiness and life satisfaction, mental health, and meaning and purpose) exhibited high intercorrelations and primarily drove the general flourishing construct. In contrast, the character and virtue and close social relationships domains were less strongly correlated with the general factor, implying a degree of conceptual divergence. These two domains also exhibited relatively high intercorrelations with each other, implying the possibility of a secondary clustering distinct from the other flourishing components. Our findings imply a culturally distinct perspective on how items within character and virtue are interpreted. Specifically, they opened the possibility that respondents distinguish between personal components (happiness and life satisfaction, mental health, meaning and purpose) and relational components (character and virtue, close social relationships) within a multidimensional well-being framework. For example, a study on meaning in life in South Korea found that meaning consists of both individual factors and group- or community-oriented factors (Park and Kwon, 2012). Furthermore, in relationship-oriented East Asian societies, tendencies such as self-regulation (e.g., delayed gratification, CV2) and the pursuit of virtue (e.g., promoting good, CV1) may be interpreted as means of aligning with others’ expectations (i.e., self-other control) and are closely tied to moral reputation (Hitokoto and Takahashi, 2021; Krys et al., 2023). These findings highlight the need for further research to clarify the role of general and domain-specific factors, particularly regarding whether personal well-being dimensions are prioritized as primary indicators of flourishing, or whether personal and relational factors interact in shaping self-assessments of well-being in Asian cultures.

The convergent and discriminant validity results suggest that both the FI and SFI align closely with the concept of good health, particularly mental health, while remaining conceptually distinct from health behaviors. The SFI extends the FI by adding financial and material stability (D6) domain, which represents the resources necessary to sustain the other five domains over time. However, supplementary analyses did not show incremental validity of the SFI over the FI in the correlation with perceived freedom to determine how to live, an external variable reflecting the ability to make life choices and enjoy life, which is often conceptualized as financial well-being (Aubrey et al., 2022). Recent discussions on financial well-being have broadened to include the role of social comparison (Sorgente and Lanz, 2019)—how individuals evaluate their financial situation in relation to others. In South Korea, perceptions of economic inequality and relative deprivation have been identified as key factors that can undermine flourishing (Lee and Han, 2024). These findings underscore the need to consider not only objective financial conditions, but also subjective perceptions of financial and material stability within specific sociocultural contexts. In the present study, the financial and material stability domain was excluded from the IRT analyses due to its empirical distinctiveness. However, this exclusion does not diminish its theoretical importance. Future research should investigate how subjective perceptions of financial and material stability vary across sociocultural contexts, particularly in settings characterized by perceived economic insecurity. Conducting targeted analyses on this domain across diverse populations may help clarify whether D6 represents a distinct facet of ‘secure flourishing’ that reflects the sustainability of well-being, and may also reveal how perceptions of financial insecurity vary with cultural context and economic conditions.

To our knowledge, this study represents the first investigation to apply multidimensional IRT analysis to the FI. Implementing the multidimensional GRM addressed violations of the local independence assumption and enabled detailed examination of item-level functioning. Specifically, items within the character and virtue domain require refinement, as they demonstrated low discrimination and a limited contribution to measurement precision in the South Korean context. The tendency toward higher-category endorsements in this domain may reflect social desirability, a response bias in which participants answer in ways perceived as socially favorable, a phenomenon commonly observed in self-report measures (VanderWeele and Johnson, 2025b). This domain comprises two items assessing tendencies to *promote good* and to *delay gratification*, reflecting cardinal virtues such as justice, prudence, temperance, and fortitude (VanderWeele, 2017). These constructs align with sociomoral norms emphasized in South Korea, where values related to social harmony and self-cultivation are central components of moral development (Lalwani et al., 2006; Takamatsu et al., 2024).

The intended meaning of ‘promoting good’ refers specifically to doing good for others and those around oneself (Lomas et al., 2025). In Korea, this resonates with a distinctive form of collectivism characterized by mutual altruism, dense social bonding, and an emphasis on “*we-ness*” in interpersonal relations (Choi and Choi, 1994; Takamatsu et al., 2024). Behaviors that promote harmonious relationships or demonstrate respect for others are widely regarded as socially desirable and morally commendable. The construct ‘delayed gratification’ similarly reflects Confucian traditions that emphasize self-cultivation, self-discipline, and the moral responsibility to regulate one’s mind and actions (Chung, 2015). Practices of self-restraint and perseverance are central to Confucian ethics and may amplify socially desirable response tendencies. Taken together, these cultural dynamics suggest that Korean respondents may have been more likely to endorse these virtue-related items in ways reflecting social desirability and sensitivity to external moral evaluation, contributing to the elevated endorsement and lower discrimination observed in this domain.

In contrast to this pattern of socially desirable overendorsement, item H2 (physical health) displayed a different culturally driven response tendency. H2 (physical health) exhibited wide threshold gaps and flattened category characteristic curves, along with a tendency for endorsement at lower response categories. Previous research has explained this pattern within a sociocultural framework, noting that East Asians, including Koreans, tend to apply higher expectations and stricter self-evaluations when assessing their health compared with Western populations (Lee and Shinkai, 2003; Choi and Miyamoto, 2022). This may reflect cultural norms emphasizing humility and modest self-presentation (Kim, 2023), in which individuals avoid extreme or overly positive responses and instead provide more conservative self-assessments (Tanaka et al., 2023). Together, these findings indicate that Korean respondents may exhibit both socially desirable overendorsement for morally valued virtues and modest, self-lowering tendencies for self-evaluative items, depending on the cultural meaning of the construct. These contrasting patterns underscore culturally specific variation in item functioning, and reinforce the need for culturally informed interpretation.

The multidimensional GRM also revealed misfits in certain items, most notably inflated discrimination parameters for HS2 (happiness) and the close social relationships items (CR1, CR2). No definitive evidence was identified to explain this issue. Given the high communality values and the similarity of response patterns between item pairs within their respective domains, these findings likely reflect redundancy or strong conceptual overlap. However, alternative explanations, including cultural response tendencies and local dependence effects, remain possible.

Given that this scale is already used extensively in international comparative research (VanderWeele et al., 2025), producing culturally nuanced yet still comparable findings requires a clearer understanding of how respondents in different cultural contexts interpret and evaluate each item. To ensure consistent interpretation across cultural groups, cognitive interviewing and qualitative probing are crucial next steps. Furthermore, when conducting domain-specific research focused on the Character and Virtue domain, there is a need to develop or supplement items that more directly reflect the sociocultural context of Korea. Researchers may consider behaviorally anchored or frequency-based item formats, which reduce evaluative pressure and cultural response bias. Complementary assessment methods, such as situational judgment tasks or vignette-based evaluations, could also enhance ecological validity by assessing virtue-related behavior in more concrete and culturally grounded scenarios.

Finally, the FI scale provided the greatest information among respondents with lower latent trait levels (i.e., those with lower flourishing), indicating that it is particularly well-suited for differentiating individuals at the lower end of the flourishing continuum (Supplementary Figure S9). At the same time, this also points to limitations in capturing individuals with very high flourishing in the Korean context. A natural extension for future research would be to develop additional items capable of discriminating among highly flourishing groups, thereby enhancing measurement precision across the full continuum of flourishing. However, given that the FI and SFI are established instruments used in large-scale international research, including the Global Flourishing Study (VanderWeele et al., 2025), opportunities for revising item content are limited. Therefore, rather than altering the scales, caution is warranted in interpreting results for respondents at the highest levels of flourishing in this cultural context.

This study had several limitations, including those discussed above. The first limitation of this study concerns the representativeness of the sample. Although the study utilized a well-established national panel designed to reflect essential demographic distributions such as age, gender, and region, and the large sample size (*n* > 1,200) provided sufficient statistical power for item-level analyses, potential self-selection bias cannot be fully excluded. Individuals with higher digital literacy, education, or motivation to participate in surveys may have been overrepresented. To include populations with more diverse socioeconomic backgrounds, future studies should adopt probability-based sampling (e.g., multistage stratified or cluster sampling) grounded in updated census frames and consider mixed-mode data collection and post-stratification weighting to enhance representativeness. Furthermore, as this study focused on examining the structural validity of the instrument using a single cross-sectional survey, longitudinal evidence of measurement stability could not be assessed. To strengthen the psychometric robustness of the scale, future research should evaluate test–retest reliability and other longitudinal indices of stability and predictive validity, providing a more comprehensive validation framework over time. Second, the scale used in this study comprised only two items per domain, thereby constraining the psychometric analyses. For example, the limited item pool hindered a full evaluation of the dominance of the general factor and the independent contributions of group factors via bifactor modeling. Similarly, the unidimensional IRT analysis violated the assumption of local independence, necessitating the application of multidimensional IRT models. However, these models were also challenged by structural limitations arising from the two-item-per-domain format, which led to parameter misfit and interpretive challenges. Despite these constraints, as noted by VanderWeele et al. (2020, 2021), short instruments are widely used in applied research, for example, the four-item purpose in life scale (Schulenberg et al., 2011) and the three-item loneliness scale (Hughes et al., 2004). While such measures inevitably carry methodological limitations, their investigation remains valuable, and the present study contributes meaningfully to this body of work.

Finally, IRT analyses are not immune to the influence of response styles or cultural tendencies (Oishi, 2006; Krys et al., 2023). Although we sought to interpret item-level characteristics with sensitivity to cultural context, such interpretations inherently require more comprehensive approaches to capture cross-cultural variation. Future studies should integrate qualitative components and expand the item pool to enable a more nuanced understanding of cultural influences on flourishing assessments.

## 5 Conclusion

This study translated the FI and SFI, comprehensive instruments that conceptualize multidimensional human flourishing, into Korean, and evaluated their psychometric validity. Both scales demonstrated strong structural validity, while also revealing specific areas that warrant refinement. In particular, the character and virtue domain requires a more culturally nuanced interpretation and closer examination of its relationship with the general flourishing construct. Furthermore, the happiness item showed potential redundancy with other domains, while the two items within the close social relationships domain exhibited substantial overlap. Given that these instruments are well-established and widely used in large-scale international research, including the Global Flourishing Study^1^, substantive modifications, although desirable from a psychometric perspective, may compromise cross-country comparability. Accordingly, we recommend interpretive caution when analyzing these domains and the complementary use of qualitative approaches, such as cognitive interviews, to examine how individual items and the overarching construct of flourishing are understood in the Korean sociocultural context. The findings of this study contribute in two important ways. First, to our knowledge, no existing instrument in Korea comprehensively assesses flourishing by uniquely integrating both health and virtue as central components. The validated Korean versions therefore provide a crucial tool for monitoring flourishing in this cultural setting. Second, by applying item response theory alongside classical test theory, this study delivers fine-grained psychometric insights into the FI and SFI, enriching understanding of their item-level functioning. Moreover, these analyses generate culturally grounded evidence on how the construct of flourishing is interpreted in Korea, thereby informing future cross-cultural research and applications.

## Data Availability

The raw data supporting the conclusions of this article will be made available by the authors, without undue reservation.

## Footnotes

1
https://globalflourishingstudy.com
