# Correction: Validating the Korean versions of the flourish index and the secure flourish index: a comprehensive psychometric approach

**DOI:** 10.3389/fpsyg.2026.1829173

**Published:** 2026-04-16

**Authors:** Jaeyoung Ha, Dorota Weziak-Bialowolska, Yoon Young Choi, Soong-nang Jang, Jieun Hwang, Sung-il Cho

**Affiliations:** 1 Graduate School of Public Health, Seoul National University, Seoul, Republic of Korea; 2 Department of Quantitative Methods and Information Technology, Kozminski University, Warsaw, Poland; 3 Human Flourishing Program, Institute for Quantitative Social Science, Harvard University, Cambridge, MA, United States; 4 Institute for Community Care and Health Equity, Chung-Ang University, Seoul, Republic of Korea; 5 Red Cross College of Nursing, Chung-Ang University, Seoul, Republic of Korea; 6 Department of Health Administration, College of Health Science, Dankook University, Cheonan, Republic of Korea; 7 Institute of Convergence Healthcare, Dankook University, Cheonan, Republic of Korea; 8 Institute of Health and Environment, Seoul National University, Seoul, Republic of Korea

**Keywords:** human flourishing, item response theory, multidimensional well-being, psychometrics, surveys and questionnaires

The Korean-translated version of the questionnaire was originally available from the authors upon request; however, it has now been made publicly available and is published as **Supplementary Data Sheet 1**.

The original version of this article has been updated.

