# Supplementary material for: Validating the Korean versions of the flourish index and the secure flourish index: a comprehensive psychometric approach
Source: Front Psychol. 2026 Feb 11;17:1694272. doi: 10.3389/fpsyg.2026.1694272 (PMC12932474; doi:10.3389/fpsyg.2026.1694272)
Supplement: Supplementary file 1 [file Supplementary_file_1.pdf]

Supplementary Material

1 Supplementary Tables and Figures

1.1 Supplementary Tables

Table S1. Characteristics of participants.

Characteristic	N (%)
Total	1,217 (100)
Sex = female (%)	594 (48.8)
Age group	
19-29	244 (20.0)
30-39	233 (19.1)
40-49	281 (23.1)
50-59	308 (25.3)
60-64	151 (12.4)
Married (%)	451 (37.1)
Having a religion (%)	444 (36.5)
Education (completed at least high school education) (%)	920 (75.6)

Table S2. Correlations and descriptive statistics of domains of flourishing

Domain	Pearson's correlation coefficients						Mean (SD)	ICC	Corrected item-total correlations	*Cronbach's α* if item deleted
	D1	D2	D3	D4	D5	D6				
D1. Happiness and life satisfaction	1.000						5.84 (2.01)	0.86	0.76	0.73
D2. Physical and mental health	0.740	1.000					5.79 (1.91)	0.64	0.75	0.74
D3. Meaning and purpose	0.737	0.714	1.000				6.27 (2.04)	0.76	0.75	0.73
D4. Character and virtue	0.407	0.449	0.510	1.000			6.77 (1.58)	0.37	0.48	0.80
D5. Close social relationship	0.537	0.534	0.550	0.451	1.000		6.28 (1.99)	0.87	0.58	0.77
D6. Financial and material stability	0.261	0.218	0.181	0.011	0.116	1.000	4.33 (2.33)	0.73	0.20	0.87

ICC, Intraclass Correlation Coefficient

Table S3. Correlations of the Flourish Index and Secure Flourish Index with external financial and material variables.

Measure	FI	SFI	HS (D1)	H (D2)	MN (D3)	CV (D4)	CR (D5)	FS (D6)
Free to determine how to live	0.631***	0.619***	0.558***	0.543***	0.575***	0.361***	0.490***	0.170***
Economic status at 12 years	0.222***	0.228***	0.179***	0.234***	0.178***	0.115***	0.183***	0.096***
Monthly personal income	0.186***	0.194***	0.153***	0.160***	0.174***	0.130***	0.134***	0.090***
Monthly household income	0.239***	0.274***	0.237***	0.217***	0.226***	0.119***	0.154***	0.211***
Perceived appropriate average monthly expenses	0.065*	0.073*	0.078**	0.035	0.077**	0.018	0.045	0.054

*$p < 0.05$, **$p < 0.01$, ***$p<0.001$

FI, Flourish index; SFI, Secure flourish index; HS; Happiness and life satisfaction; H, Health; MN, Meaning and purpose; CV, Character and virtue; CR, Close social relationships; FS, Financial and material stability.

Table S4. Results of measurement invariance tests for the Flourish Index.

Model	χ^2	DF	CFI	ΔCFI	TLI	ΔTLI	RMSEA	ΔRMSEA	SRMR
Flourish Index (FI) – Correlated traits model									
Gender									
Configural	100.857	50	0.992	-	0.986	-	0.047	-	0.016
Metric	106.793	55	0.992	0.000	0.987	0.001	0.046	-0.002	0.025
Scalar	**120.567**	60	0.991	-0.001	0.986	-0.001	0.047	0.001	0.026
Age group									
Configural	174.586	125	0.993	-	0.987	-	0.046	-	0.021
Metric	195.715	145	0.993	0.000	0.988	0.002	0.043	-0.003	0.030
Scalar	**263.256**	165	0.986	-0.007	0.981	-0.008	0.055	0.012	0.038
Marriage									
Configural	83.713	50	0.995	-	0.990	-	0.038	-	0.015
Metric	90.204	55	0.994	0.000	0.991	0.001	0.037	-0.001	0.022
Scalar	**100.921**	60	0.994	-0.001	0.991	0.000	0.038	0.001	0.023
Education									
Configural	105.137	50	0.992	-	0.985	-	0.049	-	0.018
Metric	111.189	55	0.991	0.000	0.986	0.001	0.047	-0.002	0.025
Scalar	118.253	60	0.991	0.000	0.987	0.001	0.045	-0.002	0.026
Religion									
Configural	81.106	50	0.995	-	0.991	-	0.037	-	0.015
Metric	82.772	55	0.996	0.001	0.993	0.002	0.033	-0.004	0.016
Scalar	90.456	60	0.995	0.000	0.993	0.000	0.033	0.000	0.016

DF, Degrees of Freedom; CFI, Comparative Fit Index; TLI, Tucker-Lewis Index; RMSEA, Root-Mean-Square Error of Approximation; SRMR, Standardized Root Mean Square Residual; Δ (Delta) represents differences in fit indices across sequential models (configural, metric, scalar). Subpopulations analyzed: Sex (male, female); Age groups (19–29, 30–39, 40–49, 50–59, 60–64 years); Marital status (married, unmarried); Education level (<college, ≥college); Religion (yes, no). Bold text denotes statistical significance ($p < 0.05$).

Table S5. Results of measurement invariance tests for the Secure Flourish Index.

Model	χ^2	DF	CFI	ΔCFI	TLI	ΔTLI	RMSEA	ΔRMSEA	SRMR
Secure Flourish Index (SFI) – Correlated traits model									
Gender									
Configural	142.661	78	0.991	-	0.986	-	0.042	-	0.020
Metric	148.861	84	0.991	0.000	0.987	0.001	0.040	-0.001	0.026
Scalar	**163.074**	90	0.990	-0.001	0.986	0.000	0.041	0.001	0.027
Age group									
Configural	252.991	195	0.993	-	0.988	-	0.039	-	0.027
Metric	278.037	219	0.993	0.000	0.989	0.001	0.037	-0.002	0.034
Scalar	**358.667**	243	0.986	-0.007	0.981	-0.008	0.049	0.012	0.040
Marriage									
Configural	131.135	78	0.993	-	0.988		0.038	-	0.021
Metric	136.544	84	0.993	0.000	0.989	0.001	0.036	-0.001	0.026
Scalar	**149.606**	90	0.992	-0.001	0.989	0.000	0.037	0.001	0.027
Education									
Configural	146.592	78	0.991	-	0.985	-	0.043	-	0.022
Metric	154.799	84	0.991	0.000	0.985	0.001	0.042	-0.001	0.028
Scalar	**171.476**	90	0.990	-0.001	0.985	-0.001	0.043	0.001	0.029
Religion									
Configural	125.163	78	0.994	-	0.989	-	0.036	-	0.021
Metric	125.976	84	0.994	0.001	0.991	0.002	0.033	-0.003	0.021
Scalar	134.270	90	0.994	0.000	0.991	0.000	0.032	-0.001	0.021

DF, Degrees of Freedom; CFI, Comparative Fit Index; TLI, Tucker-Lewis Index; RMSEA, Root-Mean-Square Error of Approximation; SRMR, Standardized Root Mean Square Residual; Δ (Delta) represents differences in fit indices across sequential models (configural, metric, scalar). Subpopulations analyzed: Sex (male, female); Age groups (19–29, 30–39, 40–49, 50–59, 60–64 years); Marital status (married, unmarried); Education level (<college, ≥college); Religion (yes, no). Bold text denotes statistical significance ($p < 0.05$).

Table S6. The fundamental assumptions for IRT (the Flourish Index)

Measure	Index	Result	Criterion
Uni-dimensionality			
Confirmatory factor analysis	CFI	0.775	>0.95
	TLI	0.711	>0.95
	RMSEA	0.214	<0.05
	SRMR	0.080	<0.05
Local independence			
Q3 residual correlation (unidimensional GRM)		HS1 – HS2 (0.368)<br>HS2 – MN2 (0.339)<br>CR1 – CR2 (0.785)	< |0.30|
Q3 residual correlation (multidimensional GRM)		HS1 – HS2 (0.236)	

Table S7. Monotonicity for each item for the Flourish Index.

Item	H	#ac	#vi	#vi/#ac	Maxvi	Sum	Sum/#ac	Zmax	#zsig	crit
HS1	0.58	105	1	0.01	0.03	0.03	0.0003	0.79	0	0
HS2	0.60	86	0	0.00	0.00	0.00	0.0000	0.00	0	0
H1	0.51	127	0	0.00	0.00	0.00	0.0000	0.00	0	0
H2	0.59	101	0	0.00	0.00	0.00	0.0000	0.00	0	0
MN1	0.58	103	0	0.00	0.00	0.00	0.0000	0.00	0	0
MN2	0.57	111	0	0.00	0.00	0.00	0.0000	0.00	0	0
CV1	0.42	101	2	0.00	0.09	0.09	0.0009	2.42	1	29
CV2	0.31	133	2	0.02	0.09	0.08	0.0008	0.74	0	13
CR1	0.50	114	0	0.00	0.00	0.00	0.0000	0.00	0	0
CR2	0.51	89	0	0.00	0.00	0.00	0.0000	0.00	0	0

HS1, Life satisfaction; HS2, Happiness; H1, Physical health; H2, Mental health; MN1, Meaningfulness; MN2, Purposefulness; CV1, Promoting good; CV2, Delayed gratification; CR1, Close social relationship; CR2, Satisfaction on close relationship.

Table S8. Differential item functioning (DIF) summary results across models for each comparator.

Item	Age (group)							Gender							Education						
	P-values			ΔR^2			Effect size	P-values			ΔR^2			Effect size	P-values			ΔR^2			Effect size
	$\chi^2_{1,2}$	$\chi^2_{1,3}$	$\chi^2_{2,3}$	$R^2_{1,2}$ †	$R^2_{1,3}$ †	$R^2_{2,3}$ †	$\beta_{1,2}$	$\chi^2_{1,2}$	$\chi^2_{1,3}$	$\chi^2_{2,3}$	$R^2_{1,2}$ †	$R^2_{1,3}$ †	$R^2_{2,3}$ †	$\beta_{1,2}$	$\chi^2_{1,2}$	$\chi^2_{1,3}$	$\chi^2_{2,3}$	$R^2_{1,2}$ †	$R^2_{1,3}$ †	$R^2_{2,3}$ †	$\beta_{1,2}$
HS1	0.444	0.515	0.390	0.000	0.000	0.000	0.000	0.226	0.021	0.015	0.001	0.004	0.002	0.008	0.226	0.021	0.015	0.001	0.004	0.002	0.008
HS2	0.006	0.019	0.390	0.001	0.001	0.000	0.002	0.124	0.037	0.057	0.001	0.003	0.002	0.009	0.124	0.037	0.057	0.001	0.003	0.002	0.009
H1	0.007	0.016	0.488	0.003	0.003	0.000	0.007	0.000	0.001	0.494	0.010	0.001	0.001	0.018	0.000	0.001	0.494	0.010	0.011	0.001	0.018
H2	0.023	0.026	0.336	0.001	0.001	0.000	0.005	0.002	0.009	0.446	0.004	0.004	0.001	0.001	0.002	0.009	0.446	0.004	0.004	0.001	0.001
MN1	0.001	0.003	0.142	0.002	0.002	0.000	0.003	0.010	0.000	0.003	0.003	0.007	0.004	0.005	0.010	0.000	0.003	0.003	0.007	0.004	0.005
MN2	0.954	0.368	0.404	0.000	0.000	0.000	0.000	0.247	0.004	0.002	0.002	0.006	0.005	0.000	0.247	0.004	0.002	0.002	0.006	0.005	0.000
CV1	0.854	0.714	0.158	0.000	0.000	0.000	0.000	0.000	0.000	0.187	0.025	0.028	0.003	0.010	0.000	0.000	0.187	0.025	0.028	0.003	0.010
CV2	0.012	0.019	0.424	0.004	0.005	0.001	0.009	0.603	0.601	0.450	0.002	0.004	0.002	0.007	0.603	0.601	0.450	0.002	0.004	0.002	0.007
CR1	0.051	0.141	0.212	0.002	0.002	0.000	0.005	0.016	0.024	0.241	0.006	0.009	0.003	0.016	0.016	0.021	0.241	0.006	0.009	0.003	0.016
CR2	0.050	0.143	0.745	0.002	0.002	0.000	0.005	0.027	0.010	0.058	0.005	0.009	0.004	0.013	0.027	0.010	0.058	0.005	0.009	0.004	0.013

†Nagelkerke pseudo R^2. R^2 threshold =0.035

HS1, Life satisfaction; HS2, Happiness; H1, Physical health; H2, Mental health; MN1, Meaningfulness; MN2, Purposefulness; CV1, Promoting good; CV2, Delayed gratification; CR1, Close social relationship; CR2, Satisfaction on close relationship

1.2 Supplementary Figures

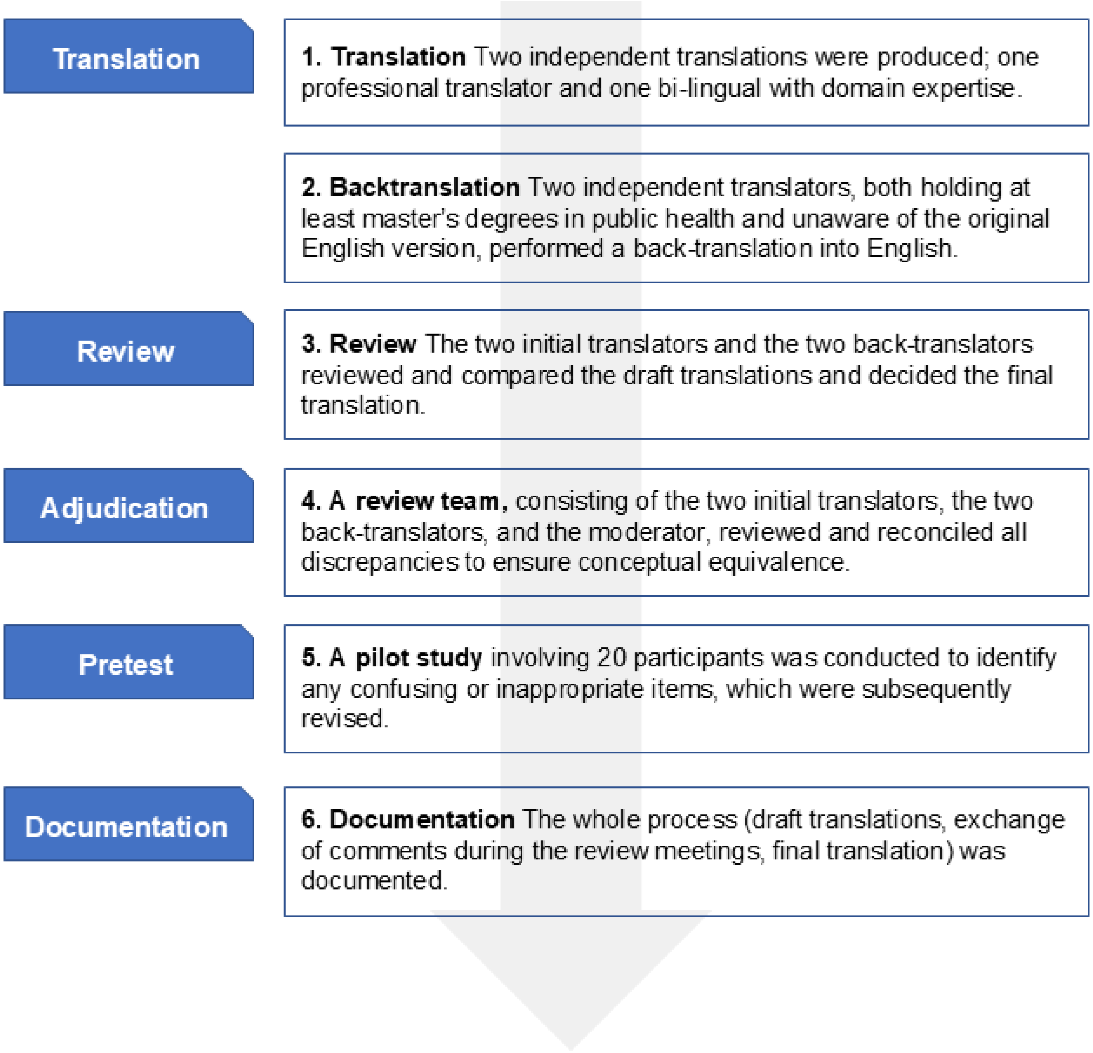

Figure S1. Translation and back-translation process of the Korean version of the Flourish Index (FI) and Secure Flourish Index (SFI).

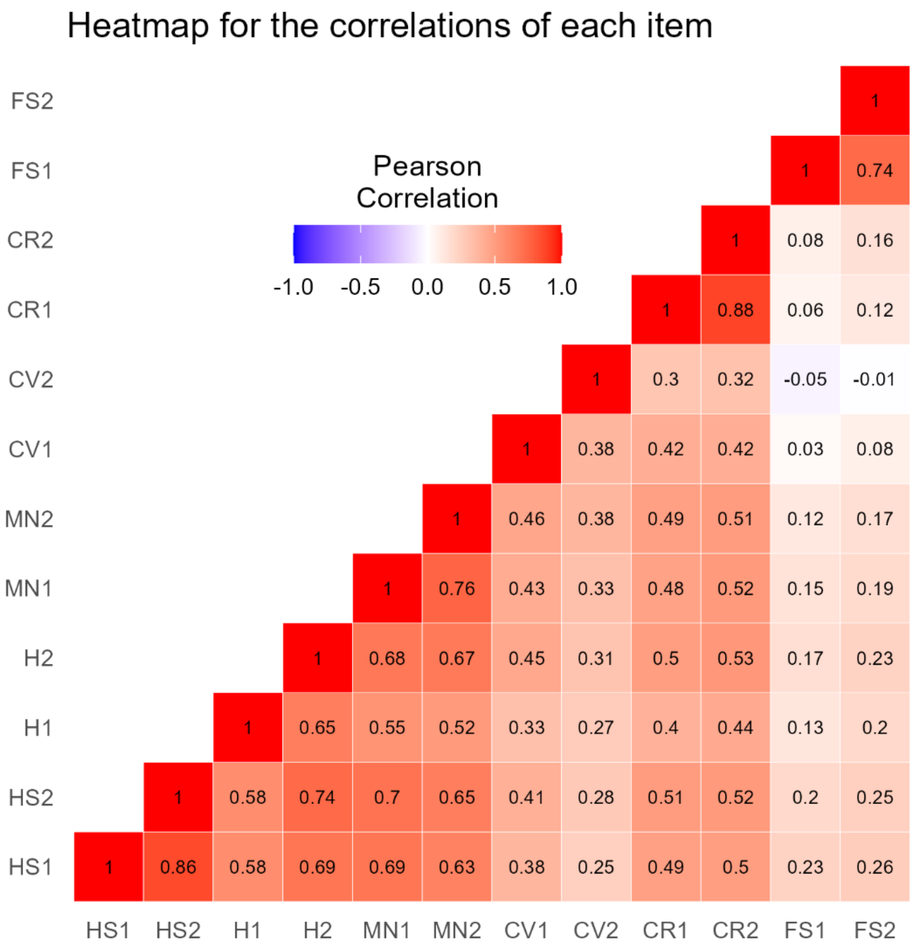

Figure S2. Heatmap of inter-item correlations across all items.

HS1, Life satisfaction; HS2, Happiness; H1, Physical health; H2, Mental health; MN1, Meaningfulness; MN2, Purposefulness; CV1, Promoting good; CV2, Delayed gratification; CR1, Close social relationship; CR2, Satisfaction on close relationship; FS1, Worry about monthly living expenses; FS2, Worry about safety, food, or housing.

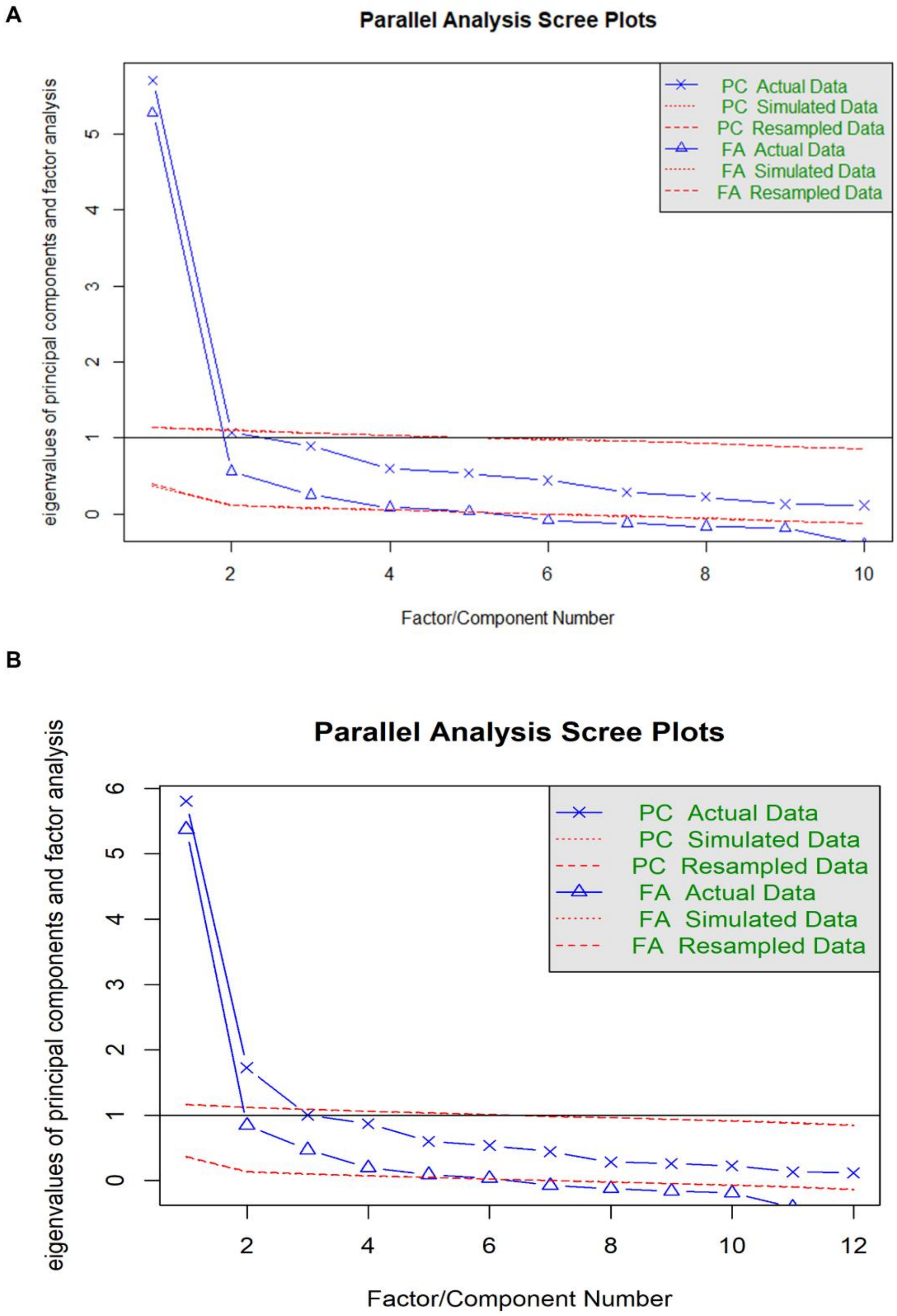

Figure S3. Parallel analysis scree plots of the (A) Flourish Index and (B) Secure Flourish Index.

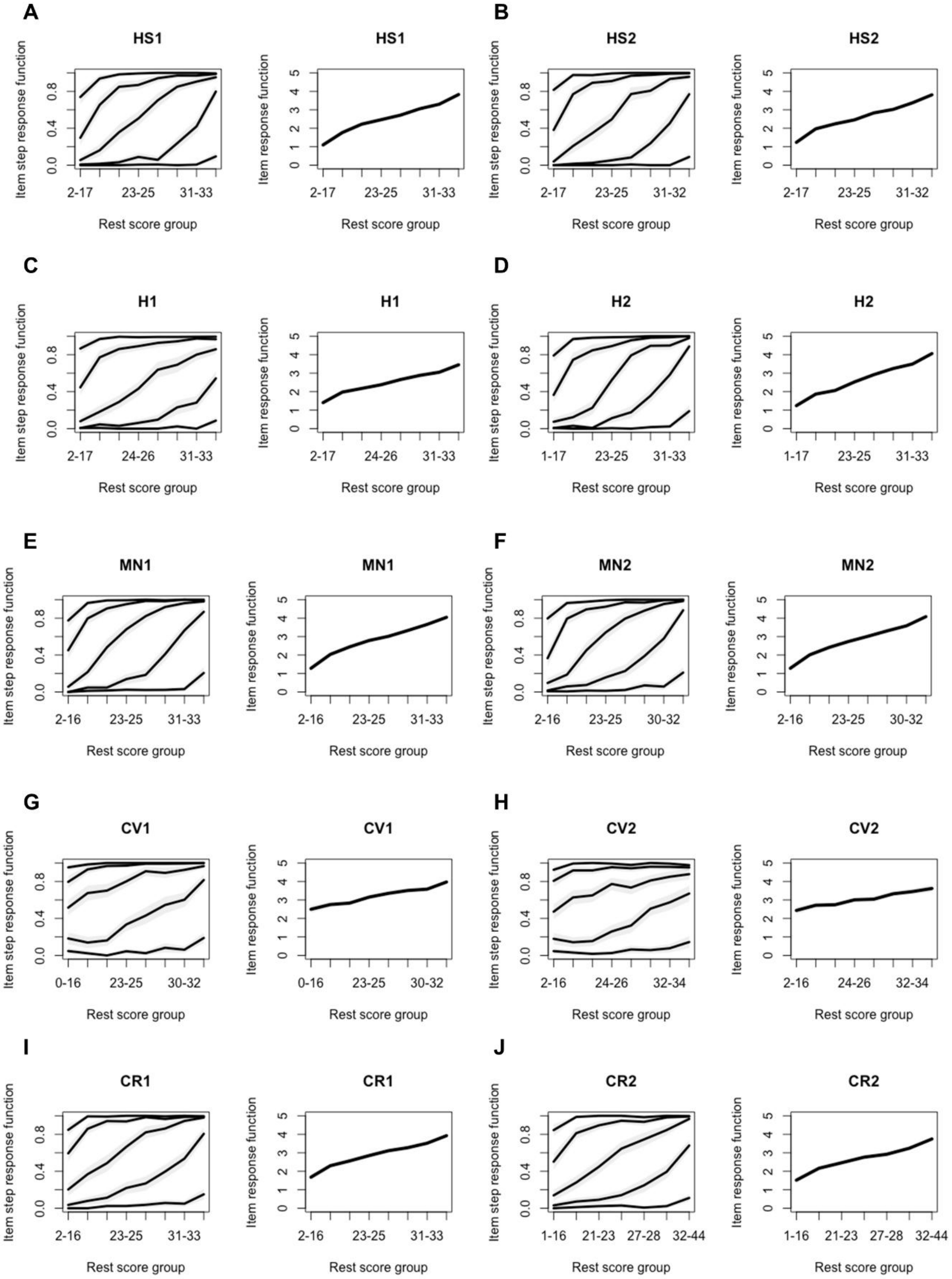

Figure S4. Monotonicity Analysis Plots Based on Mokken Scale Analysis for the Flourish Index (FI).

HS1, Life satisfaction; HS2, Happiness; H1, Physical health; H2, Mental health; MN1, Meaningfulness; MN2, Purposefulness; CV1, Promoting good; CV2, Delayed gratification; CR1, Close social relationship; CR2, Satisfaction on close relationship.

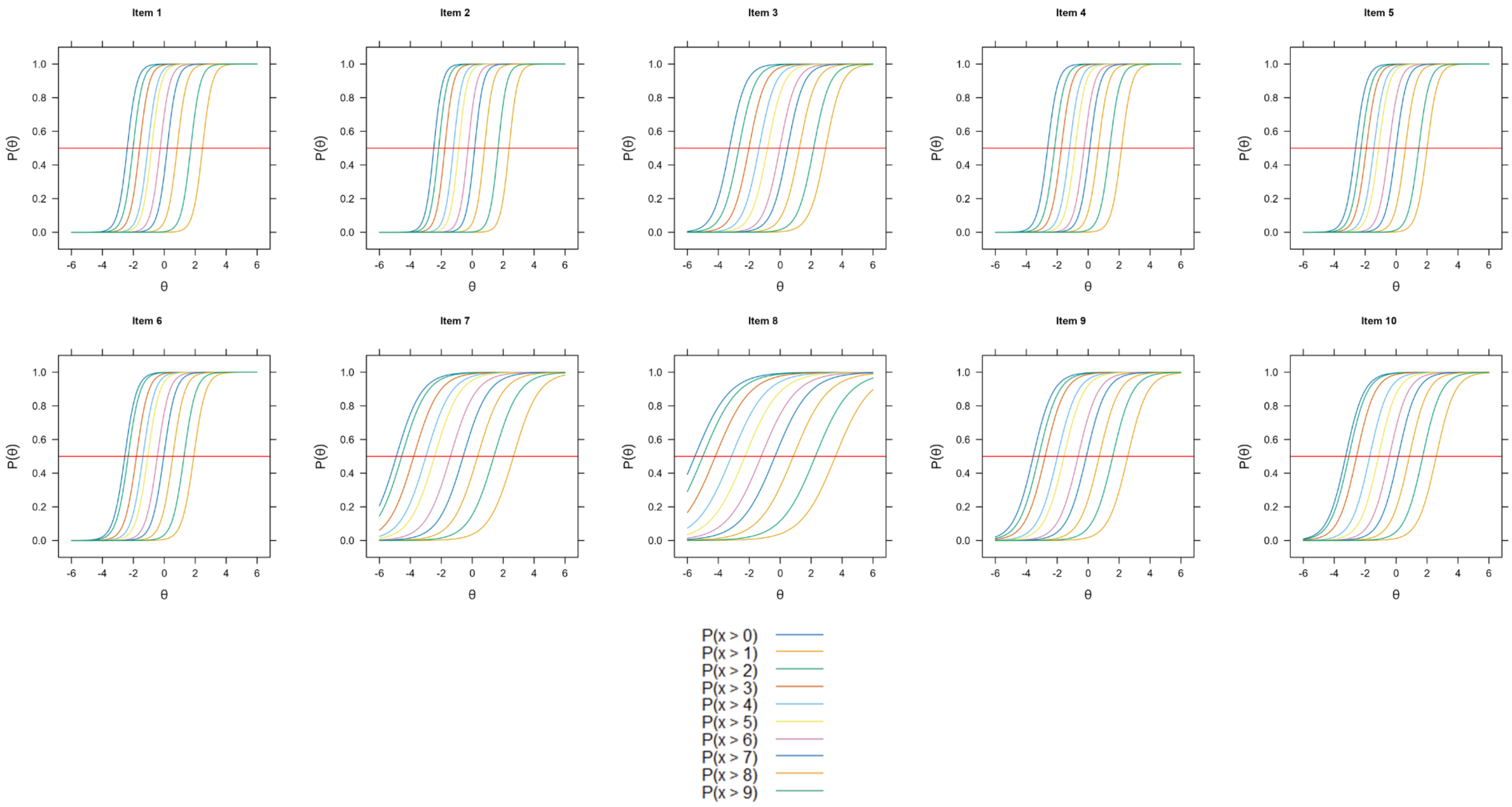

Figure S5. Boundary characteristic curves for all items of the Flourish Index.

Item 1, Life satisfaction; Item 2, Happiness; Item 3, Physical health; Item 4, Mental health; Item 5, Meaningfulness; Item 6, Purposefulness; Item 7, Promoting good; Item 8, Delayed gratification; Item 9, Close social relationship; Item 10, Satisfaction on close relationship.

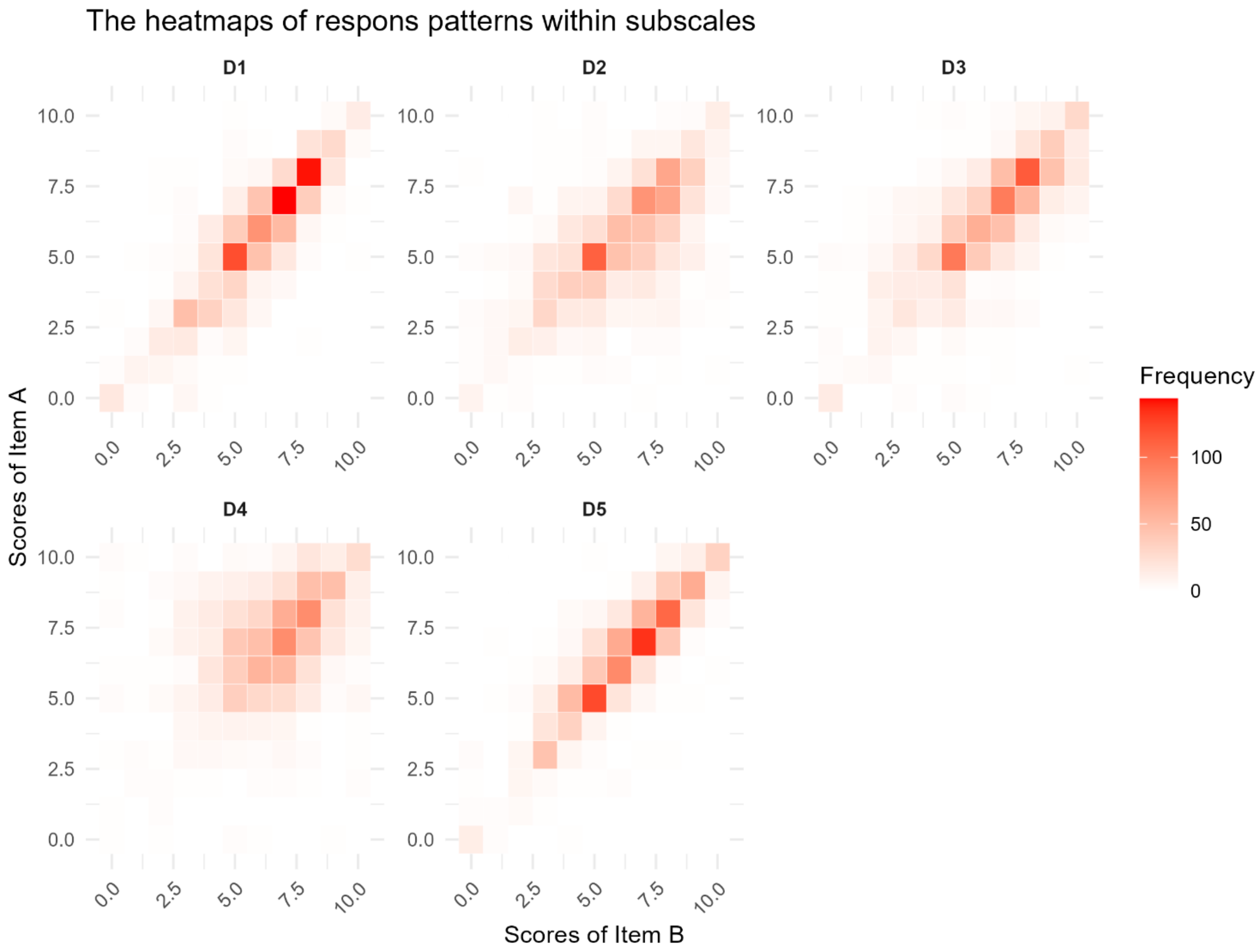

Figure S6. Heatmap visualizations of response distributions across items within each subscale (Flourish Index).

D1, Happiness and Life Satisfaction; D2, Physical and Mental Health; D3, Meaning and Purpose; D4, Character and Virtue; D5, Close Social Relationships.

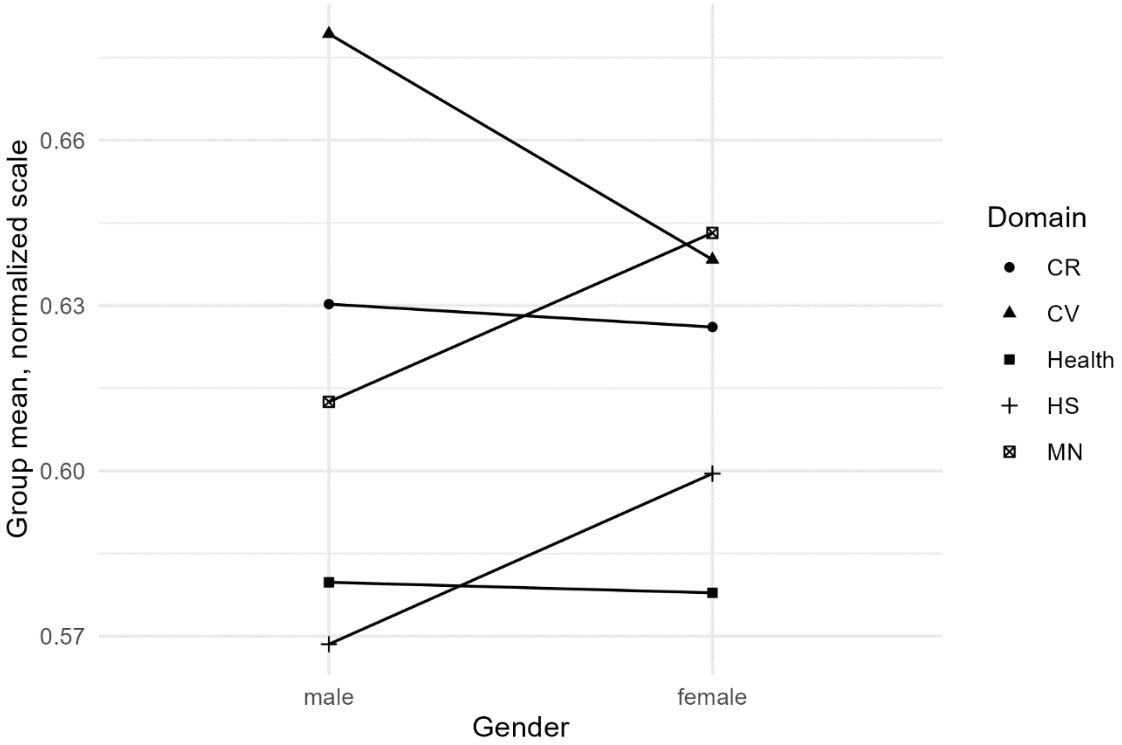

Figure S7. Differential Item Functioning by gender: domain-level group mean scores on a normalized scale.

CR, Close Social Relationships; CV, Character and Virtue; Health, Physical and Mental Health; HS, Happiness and Life Satisfaction; MN, Meaning and Purpose.

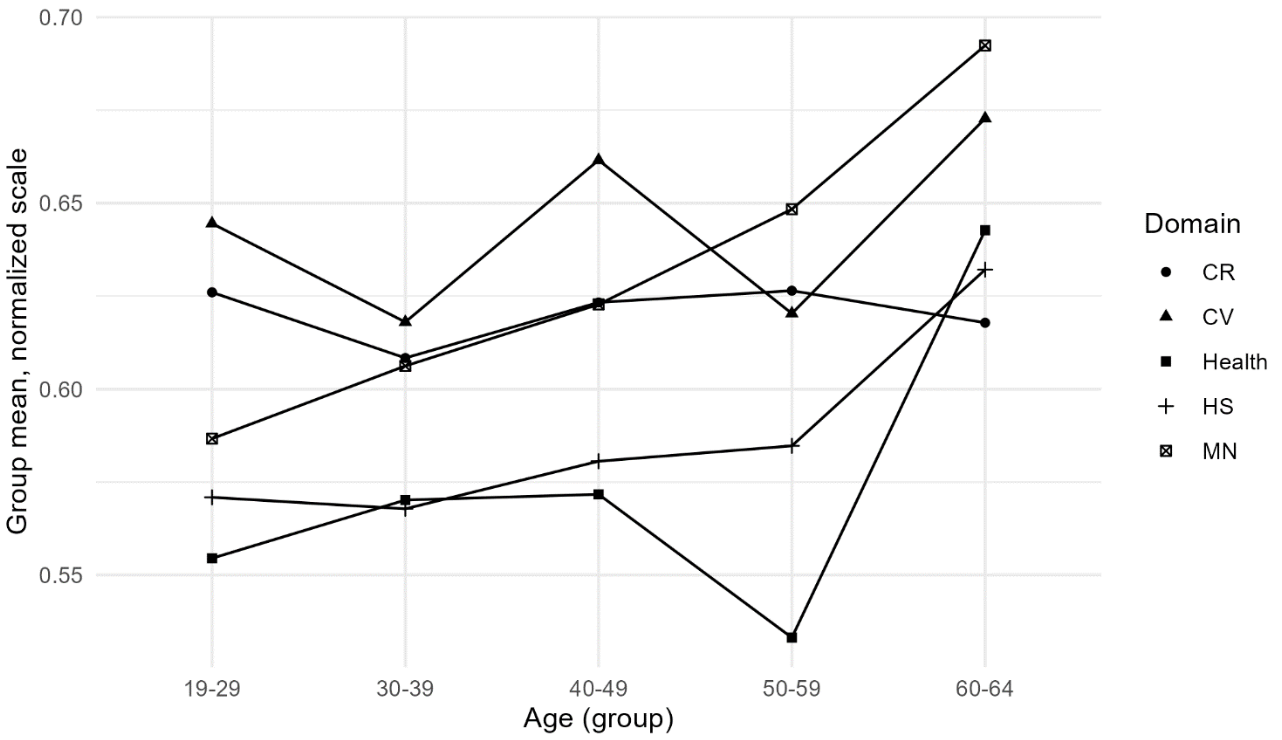

Figure S8. Differential Item Functioning by age: domain-level group mean scores on a normalized scale.

CR, Close Social Relationships; CV, Character and Virtue; Health, Physical and Mental Health; HS, Happiness and Life Satisfaction; MN, Meaning and Purpose.
