# Supplementary material for: Validating the Korean versions of the flourish index and the secure flourish index: a comprehensive psychometric approach
Source: Front Psychol. 2026 Feb 11;17:1694272. doi: 10.3389/fpsyg.2026.1694272 (PMC12932474; doi:10.3389/fpsyg.2026.1694272)
Supplement: Supplementary file 2 [file Data_Sheet_1.pdf]

Flourishing Questions (Korean translation)

The Flourish Index is composed of 10 items, with two items representing each of five dimensions: (1) Happiness and Life Satisfaction, (2) Physical and Mental Health, (3) Meaning and Purpose, (4) Character and Virtue, (5) Close Social Relationships.

The Secure Flourish Index further incorporates two items assessing (6) Financial and Material Stability, which may reflect an individual's ability to maintain flourishing over time within these five core dimensions.

※ 아래 질문에 각 항목별로 0 에서 10 사이의 숫자로 답해주세요.

Please respond to the following questions on a scale from 0 to 10:

Domain 1: Happiness and Life Satisfaction (행복 및 삶의 만족도)

1. 귀하는 요즘 자신의 삶에 대해서 전반적으로 얼마나 만족하십니까? ___

Overall, how satisfied are you with life as a whole these days?

0 = 전혀 만족하지 않는다, 10 = 완전히 만족한다

0 = Not Satisfied at All, 10 = Completely Satisfied

2. 전반적으로, 귀하는 평소에 얼마나 행복하거나 불행하다고 느끼십니까? ___

In general, how happy or unhappy do you usually feel?

0 = 매우 불행하다, 10 = 매우 행복하다

0 = Extremely Unhappy, 10 = Extremely Happy

Domain 2: Physical and mental health (신체 및 정신적 건강)

3. 귀하는 자신의 신체적 건강에 대해 어떻게 평가하시겠습니까? ___

In general, how would you rate your physical health?

0 = 매우 나쁨, 10 = 매우 좋음

0 = Poor, 10 = Excellent

4. 귀하는 자신의 정신적 건강에 대해 어떻게 평가하시겠습니까? ___

How would you rate your overall mental health?

0 = 매우 나쁨, 10 = 매우 좋음

0 = Poor, 10 = Excellent

Domain 3: Meaning and Purpose (삶의 의미와 목적)

5. 전반적으로, 귀하의 삶에서 하시는 일들이 어느 정도 가치 있다고 느끼십니까? ___

Overall, to what extent do you feel the things you do in your life are worthwhile?

0 = 전혀 가치가 없다, 10 = 매우 가치가 있다

0 = Not at All Worthwhile, 10 = Completely Worthwhile

6. 나는 내 삶의 목적을 이해한다. ___

I understand my purpose in life.

0 = 전혀 동의하지 않는다, 10 = 매우 동의한다

0 = Strongly Disagree, 10 = Strongly Agree

Domain 4: Character and Virtue (성격과 덕성)

7. 나는 어려운 상황에서도 항상 선(善)을 행하려고 노력한다. ___

I always act to promote good in all circumstances, even in difficult and challenging situations.

0 = 나에게 전혀 해당하지 않는다, 10 = 나에게 완전히 해당한다

0 = Not True of Me, 10 = Completely True of Me

8. 나는 언제나 나중의 더 큰 행복을 위해 지금 일부의 행복을 포기할 수 있다. ___

I am always able to give up some happiness now for greater happiness later.

0 = 나에게 전혀 해당하지 않는다, 10 = 나에게 완전히 해당한다

0 = Not True of Me, 10 = Completely True of Me

Domain 5: Close Social Relationships (가까운 사회적 관계)

9. 나는 내 친구 관계와 대인 관계에 만족한다. ___

I am content with my friendships and relationships.

0 = 전혀 동의하지 않는다, 10 = 매우 동의한다

0 = Strongly Disagree, 10 = Strongly Agree

10. 내 대인 관계는 내가 원하는 만큼 만족스럽다. ___

My relationships are as satisfying as I would want them to be.

0 = 전혀 동의하지 않는다, 10 = 매우 동의한다

0 = Strongly Disagree, 10 = Strongly Agree

Domain 6: Financial and Material Stability (재정 및 물질적 안정성)

11. 일반적인 한 달 생활비를 충당하는 것에 대해 얼마나 자주 걱정하십니까? ___

How often do you worry about being able to meet normal monthly living expenses?

0 = 항상 걱정한다, 10 = 전혀 걱정하지 않는다

0 = Worry All of the Time, 10 = Do Not Ever Worry

12. 귀하의 안전, 식량, 주거 문제에 대해 얼마나 자주 걱정하십니까? ___

How often do you worry about safety, food, or housing?

0 = 항상 걱정한다, 10 = 전혀 걱정하지 않는다

0 = Worry All of the Time, 10 = Do Not Ever Worry
